# Ion Channel-Targeting Toxins: Structural Mechanisms of Activation, Inhibition, and Therapeutic Potential

**DOI:** 10.3390/toxins17120579

**Published:** 2025-12-02

**Authors:** Narumi Aoki-Shioi, Shuhei Nomura, Yasuyoshi Tanaka, Shinichi Hirose

**Affiliations:** 1 Department of Medical Technology, Faculty of Fukuoka Medical Technology, Teikyo University, 6-22 Misakimachi, Omuta 836-8505, Fukuoka, Japan; nomura@fmt.teikyo-u.ac.jp; 2 Department of Pharmaceutical Care and Health Sciences, Faculty of Pharmaceutical Sciences, Fukuoka University, 19-1 8-chome, Nanakuma, Jonan-ku, Fukuoka 814-0180, Fukuoka, Japan; yasutanaka@fukuoka-u.ac.jp; 3 General Medical Research Center School of Medicine, Fukuoka University, 45-1 7-chome Nanakuma Jonan-ku, Fukuoka 814-0180, Fukuoka, Japan; hirose@fukuoka-u.ac.jp

**Keywords:** ion channel toxins, peptide toxins, pore blockade, voltage-sensor domain (VSD), gating modifier, cryo-EM, state-dependent modulation

## Abstract

Toxins as channel probes, small guanidinium alkaloids, such as tetrodotoxin and saxitoxin, canonical pore occlusion in voltage-gated Na^+^ channels. Cystine-rich peptides from spiders, scorpions, cone snails, and sea anemones, which act as pore blockers or gating modifiers targeting voltage-sensing domains. Recent structural and electrophysiological studies have identified specific binding sites on ion channels, including the S5–S6 pore loops, outer vestibule and turret regions, and S3–S4 “paddle” motifs in NaV, Kv, and CaV channels. These discrete binding epitopes are recognized by different peptide toxins, enabling isoform- and state-specific modulation; for example, μ-conotoxins bind the NaV pore, whereas charybdotoxin and agitoxin target the Kv outer vestibule. Beyond mechanistic insights, peptide toxins inspire translational strategies, including emerging therapies for retinal degenerative diseases. Photopharmacology using chemical photoswitches allows reversible, light-controlled modulation of ion channels in retinal ganglion cells without genetic manipulation or cell transplantation. Although BENAQ was discovered by small-molecule screening rather than toxin-guided design, its ion channel control demonstrates the potential of toxin-based molecular determinants for engineering synthetic compounds. This review thus integrates structural, functional, and translational perspectives, emphasizing the versatility of animal-derived peptide toxins as molecular probes and as blueprints for precision ion channel modulation in health and disease.

## 1. Introduction

Ion channels and receptors are central to numerous physiological and pathological processes, ranging from neurological disorders to cardiovascular and epithelial conditions [1]. Membrane protein dysfunction due to genetic mutations, altered regulation, or pathological modulation results in the loss of function or impaired activity, contributing to disease states [2,3]. Therapeutic strategies aimed at restoring or enhancing their function have shown promising clinical potential [2]. To design therapeutic strategies aimed at restoring or enhancing ion channel function, a detailed understanding of the fundamental properties of these channels and the structural domains underlying their activity is essential. Studies of naturally occurring bioactive compounds, including animal toxins, have provided critical insights into both channel function and the structural basis of their activity.

Despite the fact that inhibitory toxins have been extensively studied and their structural bases well characterized, the molecular mechanisms underlying toxin-mediated channel activation remain poorly understood. Activators often employ complex strategies, such as stabilizing conductive conformations or engaging voltage-sensing elements; however, only a limited number have been resolved at high resolution [4,5,6]. Additionally, while studies of ion channels and their interactions with toxins have predominantly focused on isolated channels, it is crucial to recognize that channels function within integrated cellular and tissue contexts, and their physiological outcomes are highly cell-type dependent. For example, inhibition of Kv channels may prolong action potentials in cardiac myocytes but induce tonic firing in neurons. Even within neurons, identical Kv1 isoforms can fulfill distinct functional roles depending on the neuronal subtype; for example, Kv1 channels expressed in touch-sensitive neurons exhibit properties that differ from those found in cold-sensitive sensory neurons. Such cell-type–dependent functional diversity is also reflected in the repertoire of peptide toxins targeting Kv channels, and the extensive diversity cataloged in the Kalium database (https://kaliumdb.org/, accessed on 1 November 2025) further underscores the need to consider cell-type–specific channel function when interpreting toxin actions [7]. Understanding that the functional outcomes of ion channels depend strongly on cellular and tissue context is essential for accurately interpreting the effects of toxins and their physiological or therapeutic significance.

Pharmacologically active toxins are commonly categorized as inhibitors or activators. Inhibitors suppress channel activity by blocking the conduction pathway or stabilizing non-conductive states, whereas activators enhance activity by promoting pore opening or stabilizing conductive states. The effects of these toxins on cellular excitability depend on the channel type: activation of Na^+^ or Ca^2+^ channels generally increases excitability, while activation of K^+^ channels typically promotes repolarization, thereby reducing excitability. Thus, the physiological outcome of channel modulation is highly dependent on the specific ion channel involved [8,9,10]. In receptor pharmacology, toxins and other modulators are often described as agonists or antagonists according to their interactions with ligand-binding receptors [11,12]. Agonists bind to receptor ligand sites and mimic endogenous ligands to trigger activation, whereas antagonists occupy the same or overlapping binding sites without inducing activation, thereby preventing agonist-mediated opening [11,12,13,14]. However, voltage-gated ion channels (VGICs) are primarily controlled by changes in membrane potential rather than by ligand binding; thus, the agonist–antagonist terminology is not directly applicable and may lead to conceptual confusion. For VGICs, modulatory effects are more appropriately described in terms of functional outcomes—namely, activation, inhibition, or gating modification—rather than ligand-binding relationships.

**Figure 1:**
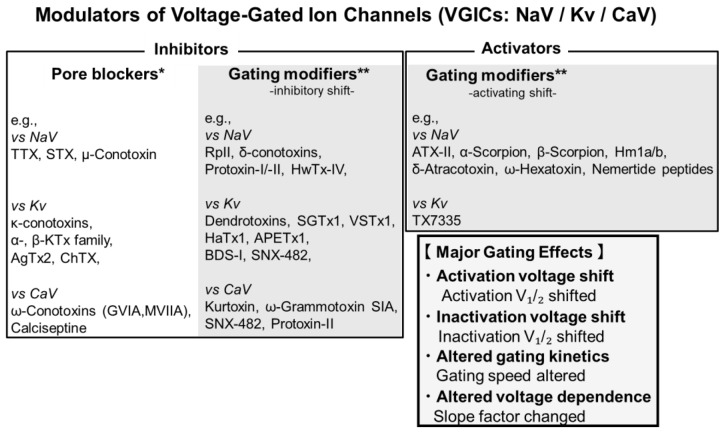
Structural and functional classification of toxin-mediated modulation of voltage-gated ion channels (VGICs: NaV, Kv, and CaV). Toxins that modulate VGICs can be grouped functionally into inhibitors and activators. Pore blockers—including TTX, STX, and μ-conotoxins—bind to the pore domain (PD; S5–S6 and the P-loop) and inhibit ion conductance by physically occluding the pore and/or stabilizing non-conductive conformations. Gating modifiers constitute a mechanistically distinct class: unlike pore blockers, which are uniformly inhibitory, gating modifiers can produce either inhibitory or activating effects depending on how they reshape voltage-dependent gating parameters. Gating modifiers—including α-scorpion toxins, δ-/κ-conotoxins, and NaV1.1 activators such as Hm1a/Hm1b—primarily interact with the voltage-sensing domain (VSD; S1–S4), often the S3b–S4 “paddle” motif, thereby altering activation and/or inactivation transitions. Major gating effects observed for gating modifiers include: (i) activation voltage shift, (ii) inactivation voltage shift, (iii) changes in gating kinetics, and (iv) changes in the voltage sensitivity (slope factor) of activation or inactivation. V_1_/_2_ denotes the membrane potential at which 50% of channels are activated or inactivated. * Central ion-conducting pore (PD: S5–S6 and P-loop) binding. ** Voltage-sensing domains (S1–S4) binding.

In addition to simple pore blocks or ligand mimicry, many toxins exert their effects by altering the gating properties of ion channels, a phenomenon referred to as gating modification [15]. VGICs (NaV, Kv, and CaV) consist of two major structural domains: the pore domain (PD), including the S5–S6 segments and P-loop that form the central ion-conducting pore, and the voltage-sensing domain (VSD; S1–S4), which controls voltage-dependent gating. Within the VSD, the S3b–S4 helix–loop region (paddle motif) serves as a key determinant of voltage sensing and a common binding site for gating-modifier toxins (Figure 1). This unified structural framework is conserved across VGIC families. This mechanism involves interactions with regulatory domains, most commonly the VSD, which shift the voltage dependence of activation or inactivation or change the kinetics of channel opening and closing [16]. Such modifications can include: (i) a negative activation shift, whereby channels open at more hyperpolarized potentials; (ii) an inactivation shift, whereby channels more readily enter or remain in non-conductive states; (iii) kinetic modulation of opening or closing rates; and (iv) changes in voltage sensitivity that alter the responsiveness to membrane potential changes. Unlike classical pore blockers, gating modifiers fine-tune the dynamic behavior of ion channels without necessarily obstructing the permeation pathway, offering a distinct and often subtler regulation mode [8,17].

In this review, we aim to consolidate the current knowledge on both activator and inhibitor toxins, with a particular focus on their structural binding modes and mechanistic principles of action. By integrating insights from structural biology, and pharmacology, we provide a framework for understanding toxin–channel interactions that extend beyond pore blocks to encompass ligand recognition and gating modifications. Our scope is restricted to natural peptides and small-molecule toxins that directly interact with ion channels; toxins that act indirectly through upstream signaling pathways or those whose effects are unrelated to ion channel modulation are excluded. Instead of offering an exhaustive catalog, we highlight representative examples that illuminate generalizable mechanistic and therapeutic principles. Ultimately, we argue that deeper structural insights into toxin-mediated activation, although still limited, will provide valuable guidance for in silico drug design and the rational development of therapeutic agents targeting channelopathies.

## 2. Classification of Ion Channel-Interacting Toxins

This section classifies animal-derived peptide toxins, excluding those from bacteria, dinoflagellates, and plants, which target voltage-gated sodium (NaV), potassium (Kv), and calcium (CaV) channels from a pharmacological perspective. Based on their mechanisms of action, these toxins can be broadly categorized as pore-targeting inhibitors and activators or gating modulators. This classification provides a systematic framework for organizing the modes of action of animal-derived peptide toxins according to their structural mechanisms, allowing for a comparison with low-molecular-weight toxins and highlighting their physiological and pharmacological significance. The following subsections summarize representative toxins for each VGIC subtype from a functional perspective. Only toxins with experimentally resolved toxin–channel complex structures are revisited in Section 3, whereas Section 2 also provides putative binding regions when structural data are unavailable.

### 2.1. Toxins Targeting NaV

NaVs are essential for the initiation and propagation of action potentials in excitable cells, including neurons and muscle fibers [18,19]. Mutations in NaV genes, known as channelopathies, reveal their essential roles in normal cellular functions and may serve as therapeutic targets. Neurological and neuromuscular forms are typically dominantly inherited and present with episodic symptoms such as seizures, migraine, ataxia, muscle stiffness, and weakness. To date, disease-associated variants have been reported in four α-subunits and one β-subunit [20,21,22,23,24]. Numerous toxins derived from invertebrates (e.g., marine cnidarians and terrestrial arthropods) and vertebrates (e.g., marine fishes and terrestrial reptiles) specifically target NaV by either blocking ion conduction or modulating channel gating properties. These toxins serve not only as potent natural agents in predator-prey interactions but also as invaluable tools in neuroscience research and drug discovery [25,26]. Studies on NaV-targeting toxins have significantly advanced our understanding of ion channel structure and function, contributing to progress in neurobiology, pharmacology, and toxinology [26,27]. Continued investigation of these natural compounds holds great promise for developing novel therapeutics, particularly for the treatment of pain and neurological disorders [26]. The structural basis and binding profiles of non-peptidic NaV toxins have been elucidated in several key studies that serve as important references for understanding their molecular mechanisms of action [17,20,28].

#### 2.1.1. Pore-Targeting Inhibition of NaV Channels

Conotoxins: A recent review [29] provided an updated classification of conotoxins and a summary of their mechanisms of action. Particularly, μ-conotoxins found in the venom of cone snails (*Conus* spp.) block NaV channels by binding to the extracellular pore region of the channel, and their blocking action is typically reversible, similarly to tetrodotoxin (TTX) and saxitoxin (STX) [30,31]. Different μ-conotoxins exhibit subtype-selective inhibition across NaV isoforms 1.1–1.8, providing valuable tools to investigate isoform-specific channel function and the functional diversity of NaV channels [29,32].

#### 2.1.2. Modulators of NaV Channel Gating

Sea Anemone Toxins: Sea anemone sodium channel toxins (NaTxs) are traditionally classified into four structural types (I–IV). Type I toxins (46–49 amino acids, three to four disulfide bonds) represent the classical long-chain NaTx family, typified by Anemonia toxin II (ATX-II) from *Anemonia sulcata*. Type II toxins, such as Radianthin peptide II (RpII) from *Heteractis crispa*, are similar in size to Type I toxins but differ in amino acid sequence and antigenicity, exhibiting species-dependent selectivity between mammalian and insect NaV channels. Type III toxins are shorter peptides (27–31 amino acids) with distinct disulfide-bond connectivity; although their effects on NaV channels are generally weaker, they can modulate other ion channels such as Kv and acid-sensing ion channels (ASICs). Representative members include Blood Depressant Substance I and II (BDS-I and BDS-II), isolated from *Anemonia viridis*. Type IV toxins have been more recently recognized as possessing structural folds distinct from those of Types I and II and may act on NaV as well as other ion channels; however, only a few representatives have been characterized to date [33,34]. Among them, ATX-II is the most extensively studied sea anemone sodium channel toxin and serves as a representative compound for electrophysiological studies. It inhibits the fast inactivation of NaV channels, thereby prolonging action potentials and enhancing neuronal excitability. To date, the high-resolution structure of the ATX-II–NaV channel complex has not been resolved [35,36].

Scorpion Venom Toxins: Scorpion venom contains α- and β-toxins that act as activator-type gating modifiers, modulating NaV channel function by targeting specific voltage-sensing domains [37]. α-Scorpion toxins (Site-3 or δ-toxins), from species such as *Leiurus quinquestriatus* and *Androctonus* spp., bind the extracellular S3–S4 loop of domain IV (VSD-IV), stabilizing the voltage sensor, delaying fast inactivation, and prolonging channel opening, thereby enhancing Na^+^ influx and cellular excitability [38]. The S3–S4 paddle motif, comprising the C-terminal portion of S3 (S3b), the S3–S4 loop, and the N-terminal portion of S4 (S4a), is essential for sensing membrane potential changes and coupling them to channel gating. Binding of α-toxins to this motif alters voltage sensitivity without blocking the pore. Functional studies and mutagenesis have identified key residues mediating these effects, although high-resolution structural complexes are not yet available [39,40,41]. β-Scorpion toxins target the paddle motif in domain II, trapping it in an activated state and causing a hyperpolarizing shift in activation. Some require a brief depolarizing pre-pulse, whereas others such as beta-toxin VII from *Centruroides suffusus suffusus* (TsVII) and toxin 1 from *Tityus zulianus* (Tz1), can act without it, possibly owing to interactions beyond Domain II [37,42]. These two classes exemplify the distinct, domain-specific mechanisms by which scorpion toxins regulate NaV channel gating.

Spider Venom Toxins: Twelve families of spider venom peptides targeting NaV channels are identified and classified. These families are distinguished by their primary amino acid sequences and arrangement of disulfide bonds, forming various structural motifs. Structural diversity is correlated with the ability of peptides to modulate NaV channel activity, highlighting the evolutionary adaptation of these toxins to interact with specific ion channel subtypes [43]. Spider venom peptides primarily target voltage-gated sodium channels by interacting with VSD, thereby modulating channel activation and inactivation. These toxins, which often adopt an inhibitory cystine knot (ICK) fold, provide valuable tools for studying channel gating mechanisms and serve as potential templates for developing novel therapeutics for neurological disorders [44,45,46].

Venoms from spiders, including δ-atracotoxins from *Atrax robustus*, act as gating modifiers of NaV channels. δ-Atracotoxins interfere with the normal fast inactivation process of these channels (NaV1.1–NaV1.7), causing sodium ions to continue entering the neuron for longer than usual. This prolonged depolarization leads to excessive neuronal firing, which can trigger muscle spasms and potentially lethal neurotoxicity. Similarly, ω-hexatoxins alter the movement of the voltage sensor in NaV channels (e.g., NaV1.2/NaV1.6), slowing the onset of inactivation and extending the duration of action potentials. The resulting sustained electrical activity further enhances neuronal excitability and contributes to severe envenomation symptoms [47,48,49].

Hm1a and Hm1b are gating-modifier peptides derived from the tarantula *Heteroscodra maculata*, consisting of 35 and 34 amino acids, respectively. Both peptides contain three disulfide bridges and adopt a Knottin-type (cystine knot) fold. As selective NaV1.1 activators, Hm1a and Hm1b bind to the S3–S4 and S1–S2 loops of the VSD-IV of NaV1.1 channels, inhibiting channel inactivation and thereby increasing neuronal excitability [50]. Protoxin-I (ProTx-I), also known as β/ω-theraphotoxin-Tp1a, is a 35-residue ICK peptide from the venom of the Peruvian green velvet tarantula (*Thrixopelma pruriens*), characterized by a compact disulfide-stabilized fold that enables high-affinity interaction with NaV voltage-sensor domains [51]; its specific channel-binding mechanism is described later in Section 3.1.2. Huwentoxin-IV (HwTx-IV) is an ICK peptide from the Chinese bird spider *Ornithoctonus huwena*, possessing a disulfide-rich compact fold typical of NaV-targeting gating modifiers [52]. Its detailed structural interactions with NaV1.7 are presented in Section 3.1.2. 

Worm Toxins: Nemertide peptides (α-1 to α-7) are gating-modifier peptides derived from the bootlace worm (*Lineus longissimus*), each consisting of 31 amino acids. All peptides contain three disulfide bridges and adopt a Knottin-type (cystine knot-like) fold. They selectively modulate NaV of crustaceans and insects, such as the green crab (*Carcinus maenas*) NaV, by inhibiting channel inactivation and thereby prolonging action potentials [53]. α-2 to α-7 have also been synthesized and evaluated for activity against NaV channels, including cockroach BgNaV1 and mammalian NaV1.1–1.8. α-1 additionally shows weak activity on mammalian NaV1.1, 1.4, 1.5, and 1.6, exhibiting approximately 100-fold selectivity for insect NaV channels over mammalian ones. The potency varies among the peptides, with α-6 showing particularly strong activity against NaV1.1 (EC_50_ value 7.9 nM) while all α-nemertides exhibit preferential activity toward BgNaV1 [54].

### 2.2. Toxins Targeting Kv

Kv channels are integral to the regulation of membrane potential and action potential repolarization in excitable cells. Functional abnormalities in Kv channels have been implicated in several neurological and psychiatric disorders, such as epilepsy [55,56], schizophrenia, and cognitive impairment. Neuronal hyperexcitability or hypoexcitability is often observed in these disorders, indicating that the balance of neural signaling may be disrupted [57]. Various toxins derived from invertebrates such as scorpions, snakes, and cone snails, as well as from vertebrates, have evolved to target these channels by either inhibiting or enhancing their activity [58,59,60,61]. The extensive diversity of peptide toxins targeting Kv channels is comprehensively cataloged in the Kalium database (https://kaliumdb.org/, accessed on 1 November 2025), which provides a curated resource of natural, artificial, and labeled polypeptides acting on potassium channels [7]. These toxins serve as valuable tools for dissecting the structure–function relationships of Kv channels and have potential therapeutic applications in neurology.

#### 2.2.1. Pore-Targeting Inhibition of Kv

Conotoxins: Certain conotoxins, such as κ-conotoxins derived from cone snails (*Conus* spp.), act as inhibitors of Kv channels. These toxins interact with the channel pore region, blocking ion flow and impairing synaptic transmission, leading to neurotoxicity and paralysis in affected organisms [62,63].

α- and β-Potassium Channel Toxins (KTx): Scorpion-derived KTx are small peptides that inhibit Kv channels. The α-KTx family comprises 23–42 amino acid peptides with three disulfide bridges that primarily block Kv1 channels, leading to increased neuronal excitability. In contrast, the β-KTx family consists of larger peptides (45–75 amino acids) that not only inhibit Kv channels, such as Kv1.3, but also display antimicrobial and immunomodulatory activities, linking ion channel blockade with host-defense modulation [64]. Toxins of the α-KTx family, such as Ts6, Ts7, Ts9, Ts15, and Ts16 from the Brazilian yellow scorpion (*Tityus serrulatus*), act as pore blockers of Kv channels. By inhibiting K^+^ conductance, they prolong depolarization, leading to increased neuronal excitability and neurotoxic effects [65,66]. In contrast, members of the β-KTx family, including Ts8, Ts19, and Ts19 Frag-I/II, inhibit Kv channels such as Kv1.3. Most β-KTx toxins inhibit Kv channels via a pore-blocking mechanism similar to α-KTx toxins, although their larger size and additional functional properties, such as antimicrobial or immunomodulatory activities, distinguish them from the α-KTx family [67]. Agitoxin-2 (AgTx2), a 38-residue peptide from *Leiurus quinquestriatus* venom, selectively blocks Shaker-type Kv1.1 and Kv1.3 channels by binding to the extracellular vestibule, thereby occluding K^+^ permeation and modulating cellular excitability [68,69].

Charybdotoxin (ChTX): ChTX, a 37-residue peptide isolated from the venom of the scorpion *Leiurus quinquestriatus hebraeus*, is a classic pore-blocking inhibitor of Kv channels. It potently inhibits Kv1.1, Kv1.3, and large-conductance Ca^2+^-activated K^+^ (BK) channels by binding to the external vestibule of the pore domain. Structural and electrophysiological studies have demonstrated that a critical lysine residue (Lys27) of Charybdotoxin penetrates the selective filter entrance, physically occluding the ion conduction pathway without perturbing the channel gating machinery [70,71]. Although the high-resolution structure of the toxin–channel complex has not been determined, the structure of the isolated toxin has been resolved (PDB ID: 2CRD), providing valuable insights into the molecular determinants of pore blockade.

Maurotoxin: Maurotoxin, a 34-residue peptide containing four disulfide bridges, is derived from the venom of the scorpion *Scorpio maurus palmatus*. Similar to Charybdotoxin, Maurotoxin functions as an outer pore blocker of Kv channels, primarily targeting Kv1.2 and Kv1.3 subtypes. Its inhibitory mechanism involves the insertion of a conserved lysine residue (Lys23) into the outer pore region, where it interacts with residues surrounding the selectivity filter to prevent K^+^ permeation [72]. Structural data of the isolated toxin (PDB ID: 1TXM [73]) have revealed a compact α/β scaffold stabilized by its disulfide pattern, which underlies its high stability and strong affinity toward Kv channels, although the toxin–channel complex structure remains unresolved [72].

#### 2.2.2. Modulators of Kv Channel Gating

Dendrotoxins: Dendrotoxins are 57–60 amino acid peptides stabilized by three disulfide bridges that belong to the Kunitz-type peptide family and are found in the venom of mamba snakes (*Dendroaspis* spp.). Four homologues (α-, β-, γ-, and δ-dendrotoxins) were identified in the same venom, each selectively targeting members of the Kv1 subfamily of voltage-gated potassium channels, particularly Kv1.1, Kv1.2, and Kv1.6, with differing affinities [74,75]. They bind to the outer vestibule of Kv1 voltage-gated potassium channels, with a critical lysine residue inserted into the outermost site of the selectivity filter, thereby partially obstructing ion conduction. This inhibition prolongs open-state depolarization of the channel, enhancing neuronal excitability and leading to sustained synaptic transmission. In envenomated animals, this mechanism can result in persistent depolarization, convulsions, or muscle spasms [75,76].

*Scodra griseipes* toxin 1 (SGTx1): SGTx1 is a 34 amino acid peptide toxin isolated from the venom of the tarantula *Scodra griseipes*. It belongs to the ICK toxin family and is characterized by a compact disulfide-bonded fold that stabilizes its β-sheet structure. SGTx1 specifically targets the VSD of Kv2.1 and related channels, binding to the S3b–S4a paddle motif [77]. Functional and mutagenesis studies have demonstrated that SGTx1 shifts the voltage dependence of activation toward more depolarized potentials, thereby inhibiting channel opening through gating modifications rather than pore occlusion [78]. The three-dimensional nuclear magnetic resonance (NMR) structure of SGTx1 was resolved (PDB ID: 1LA4), revealing a canonical ICK fold [79]. However, the high-resolution structure of the SGTx1–Kv channel complex remains to be determined. Computational and spectroscopic mapping suggested that the hydrophobic and basic residues on the surface of the toxin interact with the lipid-exposed paddle motif of VSD, consistent with its gating-modifier mechanism.

Voltage-Sensor Toxin 1 (VSTx1): VSTx1 is a 36 amino acid peptide toxin derived from the Chilean spider *Grammostola spatulata*. Like SGTx1, it belongs to the ICK family and acts as a gating modifier of voltage-gated K^+^ channels. VSTx1 selectively targets KvAP- and Kv2-like channels by binding to the VSD, particularly by interacting with the S3b–S4a paddle region [77]. Functional assays and fluorescence mapping have shown that VSTx1 stabilizes the resting conformation of the VSD, thereby shifting activation to more depolarized voltages and reducing the channel opening probability. The NMR solution structure of VSTx1 was determined (PDB ID: 1S6X [80]) and exhibited a canonical ICK motif. However, the high-resolution structure of the VSTx1–channel complex remains unresolved. Molecular dynamics and biophysical data indicate that VSTx1 partitions partially into the lipid bilayer, where it interacts with the voltage-sensor paddle motif at the protein–lipid interface, modulating voltage sensitivity through local electrostatic and hydrophobic interactions [81].

Hanatoxin (HaTx1): HaTx1 is a 42 amino acid peptide derived from the venom of the tarantula *Grammostola spatulata*. It acts as a gating-modifier inhibitor of Kv2.1 and Kv2.2 channels, binding to the S3b–S4a paddle motif of the VSD. HaTx1 shifts the voltage dependence of activation toward more depolarized potentials, thereby stabilizing the closed state of the channel and reducing the probability of channel opening. Functional and mutagenesis studies indicate that HaTx1 interacts primarily with residues in the S2 and S3 helices as well as the S3b–S4a paddle motif, consistent with its role as a VSD-targeting gating modifier rather than a pore blocker. Although the NMR structure of HaTx1 alone has been resolved, the high-resolution structure of the HaTx1–Kv channel complex has not yet been determined [15].

SNX-482: although SNX-482, a 41 amino acid peptide from the African tarantula *Hysterocrates gigas*, has been reported to affect Cav channels (detailed below), the primary focus here is its interaction with voltage-gated potassium channels. SNX-482 potently inhibits A-type currents mediated by Kv4 family channels: heterologous expression studies demonstrate blockade of Kv4.3 with an IC_50_ of <3 nM and inhibition of Kv4.2 at higher concentrations [82]. Electrophysiological data suggest that SNX-482 acts as a gating modifier rather than a simple pore occluder: at sub-saturating concentrations, it produces a depolarizing shift in the voltage-dependence of activation and slows activation kinetics of Kv4.3. These effects are consistent with interactions that may alter voltage-sensor movement or coupling between the sensor and the activation gate, although the precise molecular contacts remain to be determined. The inhibition persists when extracellular Ca^2+^ is replaced with Co^2+^, supporting a direct action on Kv channels rather than an indirect consequence of altered calcium influx. To date, no high-resolution structure of an SNX-482–Kv channel complex has been reported, and the exact binding site(s) within the pore or the voltage-sensing domain remain unknown. Consequently, state-dependent binding (open/closed/inactivated) and the detailed mechanism of action remain open questions for future mutational and structural studies.

Anthopleurin-like Peptide Toxin 1 (APETx1): APETx1 is a 42 amino acid peptide isolated from the venom of the sea anemone *Anthopleura elegantissima*. It functions as a gating-modifier inhibitor of the human ether-à-go-go-related gene (hERG) (Kv11.1) channel by binding to the S3–S4 linkers of the VSD. APETx1 shifts the activation thresholds toward more positive potentials, thereby stabilizing the closed state and reducing the channel opening probability [83]. Structural and mutagenesis studies have identified key interactions between APETx1 residues (e.g., Y5, Y32, F33, K8, and K18) and the S3b–S4 region of hERG, consistent with its gating-modifying mechanism [84,85]. Although the structure of APETx1 has been solved (PDB ID: 1WQK), the high-resolution structure of the APETx1–hERG channel complex remains unresolved [86].

Blood Depressing Substance I (BDS-I) toxin: BDS-I is a 43 amino acid peptide isolated from the sea anemone *Anemonia sulcata*, belonging to the sea anemone type III toxin family. It selectively targets members of the Kv3 subfamily, particularly Kv3.4, by modifying the voltage-dependent gating rather than occluding the pore [87]. Electrophysiological analyses have shown that BDS-I shifts the voltage dependence of activation toward more depolarized potentials and slows activation and inactivation kinetics, which is consistent with its role as an inhibitory gating modifier rather than a pore blocker [88]. Although the NMR structure of BDS-I has been determined (PDB ID: 2BDS), the high-resolution structure of the BDS-I-Kv channel complex remains to be resolved, and its precise binding interface within the VSD remains undetermined.

Other Kv Channel Activators, TX7335 is a 63 amino acid peptide toxin isolated from the venom of *Dendroaspis angusticeps* (Eastern Green Mamba) and is characterized by a three-finger toxin scaffold stabilized by four disulfide bonds. Unlike classical pore-blocking toxins, TX7335 enhances the activity of KcsA-type potassium channels by allosterically inhibiting channel inactivation. Electrophysiological studies using artificial bilayer membranes have demonstrated that TX7335 increases channel opening probability and mean opening time in a concentration-dependent manner. This novel mode of action suggests that TX7335 functions as a channel activator, providing new insights into potassium channel regulation and potential avenues for therapeutic development targeting excitable tissues [89]. However, the precise molecular mechanism through which TX7335 binds to this channel remains to be elucidated.

Some, including certain peptides and small molecules, promote the opening of Kv channels, thereby increasing K^+^ efflux and facilitating membrane repolarization. This action generally reduces neuronal excitability and helps prevent excessive firing, although the detailed molecular mechanisms remain poorly understood.

### 2.3. Toxins Targeting CaV

CaV are crucial for regulating the influx of calcium ions into cells, particularly in excitable cells such as neurons, muscle fibers, and endocrine cells. These channels play pivotal roles in neurotransmitter release, muscle contraction, and hormone secretion. Functional abnormalities in CaV channels are increasingly being recognized as critical contributors to a wide spectrum of human diseases. Pathogenic variants in CaV channel genes are associated with developmental and epileptic encephalopathies [90], episodic ataxia, familial hemiplegic migraine [91], and cardiac channelopathies, including Timothy syndrome, Brugada syndrome, and long- and short-QT syndromes [92,93]. These disorders typically arise from gain- or loss-of-function alterations in channel gating, inactivation, or trafficking, ultimately leading to abnormal calcium influx and consequent disturbances in neuronal excitability, synaptic transmission, muscle contraction, and cardiac action potential dynamics. Within this context, naturally occurring toxins and pharmacological modulators that target CaV channels have emerged as indispensable tools for elucidating channel structure–function relationships and hold promise for the development of novel therapeutic strategies.

#### 2.3.1. Pore-Targeting Inhibition of CaV

ω-Conotoxins (e.g., GVIA, MVIIA/del-Conotoxin): ω-Conotoxins, such as GVIA and MVIIA (also termed ω-conotoxin MVIIA or ziconotide), are derived from *Conus* species and typically consist of 25–30 amino acids with 3–4 disulfide bridges forming a cysteine knot motif, optimized for binding the extracellular pore region of N-type voltage-gated calcium channels (CaV2.2) [19,94]. These toxins are unique among conotoxins in that they specifically act as high-affinity pore blockers of CaV channels, whereas other conotoxin families (e.g., μ-, δ-, α-, κ-, ι-) target NaV, Kv, or nAChR channels and often act via gating modulation or competitive antagonism rather than direct pore occlusion [1,3]. These toxins bind with high affinity to the extracellular vestibule and the pore-forming region constituted by the S5–S6 helices of PDIII (the pore domain of domain III), thereby directly occluding the ion conduction pathway. In contrast, gating-modifier toxins target the S3–S4 paddle motif within VSDs, thereby directly occluding the ion conduction pathway [19,94]. This pore-blocking mechanism effectively suppresses presynaptic Ca^2+^ influx, leading to the potent inhibition of neurotransmitter release and the well-established underlying analgesic properties [95]. Importantly, unlike gating modulators that alter the probability of channel opening or the kinetics of activation and inactivation, ω-conotoxins act as classical pore blockers because they directly prevent calcium permeation without substantially modifying channel gating.

Sea Anemone Toxins (e.g., *Anthopleura* spp.): Certain peptide toxins derived from sea anemones, such as those from the genus *Anthopleura*, have been shown to modulate the activity of voltage-gated ion channels, including CaV channels. Peptides such as Hcr 1b-2 from *Heteractis crispa* exhibit an inhibitory effect on T-type CaV channels (e.g., CaV3.3), thereby reducing Ca^2+^ influx at presynaptic terminals and attenuating neurotransmitter release. This action may lead to the modulation of neuronal excitability, depending on the cellular context and the complement of target channels [96]. Although these toxins are widely used as pharmacological tools in neurophysiological studies, the precise structural mechanisms underlying their interactions with CaV channels remain poorly understood.

#### 2.3.2. Modulators of CaV Channel Gating

Kurtoxin: Kurtoxin, a 63-residue peptide isolated from the venom of the scorpion *Parabuthus transvaalicus*, adopts a CSαβ motif consisting of a single α-helix, three β-strands, and multiple turns, which are stabilized by four disulfide bridges and underlie its selective gating-modifying activity on T-type (CaV3.x) and L-type (CaV1.x) channels (state-dependent inhibition or modulation). To date, high-resolution structures of toxin–CaV1 or toxin–CaV3 complexes are largely lacking (with functional evidence available but limited structural confirmation) [97,98,99]. As a gating modifier, kurtoxin slows down both activation and inactivation kinetics and shifts the voltage dependence of channel gating toward more depolarized potentials, thereby reducing peak calcium currents [98]. This inhibitory modulation of T-type channels can attenuate calcium influx at presynaptic terminals, potentially decreasing neuronal excitability and altering synaptic transmission [99,100]. Thus, it serves as a valuable pharmacological tool for elucidating the functional roles of T-type calcium channels in excitable cells.

Spider Toxins (ω-Grammotoxin SIA, SNX-482): Peptide toxins isolated from the venoms of spiders such as *Grammostola spatulata* and *Hysterocrates gigas* were identified as gating modifiers of CaV channels [101]. ω-Grammotoxin SIA, derived from *Grammostola spatulata*, preferentially binds to the closed states of P/Q-type (CaV2.1) and N-type (CaV2.2) calcium channels, shifting their activation to more depolarized voltages and thereby reducing calcium influx and neurotransmitter release. This inhibitory effect on the calcium channels may lead to decreased neuronal excitability [102,103]. In contrast, SNX-482 selectively inhibits R-type (CaV2.3) calcium channels by interacting with voltage-sensing Domains III and IV, disrupting activation gating and leading to a pronounced reduction in calcium currents in central neurons [104,105]. Although its NMR structure has been resolved, the toxin–channel complex remains unavailable. Electrophysiological and mutagenesis studies suggest that binding to the S3–S4 extracellular loop of domain III likely impedes voltage-sensor activation [104,105]. These toxins serve as valuable tools for dissecting the structure–function relationships of voltage-gated calcium channels and have potential therapeutic applications in neurology.

Protoxin-II (ProTx-II): Protoxin-II, a 30-residue disulfide-rich peptide from the tarantula *Thrixopelma pruriens*, is a selective gating modifier of NaV1.7 channels, shifting activation to more depolarized potentials and inhibiting channel opening [106,107]. Additionally, ProTx-II can modulate CaV1.2 and CaV3.2 channels via a similar depolarizing shift in the activation voltage, although its primary pharmacological target remains NaV1.7 [108,109]. This cross-family voltage-sensor modulation makes ProTx-II a valuable tool for studying gating mechanisms across the sodium and calcium channel families [110].

## 3. Structural Analysis of Toxin and Ion Channel Interactions

Recent cryo-EM studies have revealed toxin–channel complexes at physiologically relevant conformations. In this section, we examine the binding interactions between biologically derived peptide/protein toxins and their target ion channels using cryo-EM structural analysis. The reported binding modes of ion channels and receptor inhibitors are broadly classified into two major categories: (1) physical blockade of the channel pore, which primarily results in inhibitory activity, and (2) conformational modulation associated with channel gating, which can produce either inhibitory or activating effects. Herein, we focus on gaining structural insights into the latter binding mode, which is directly linked to the ON/OFF regulation of channel functions.

**Figure 2:**
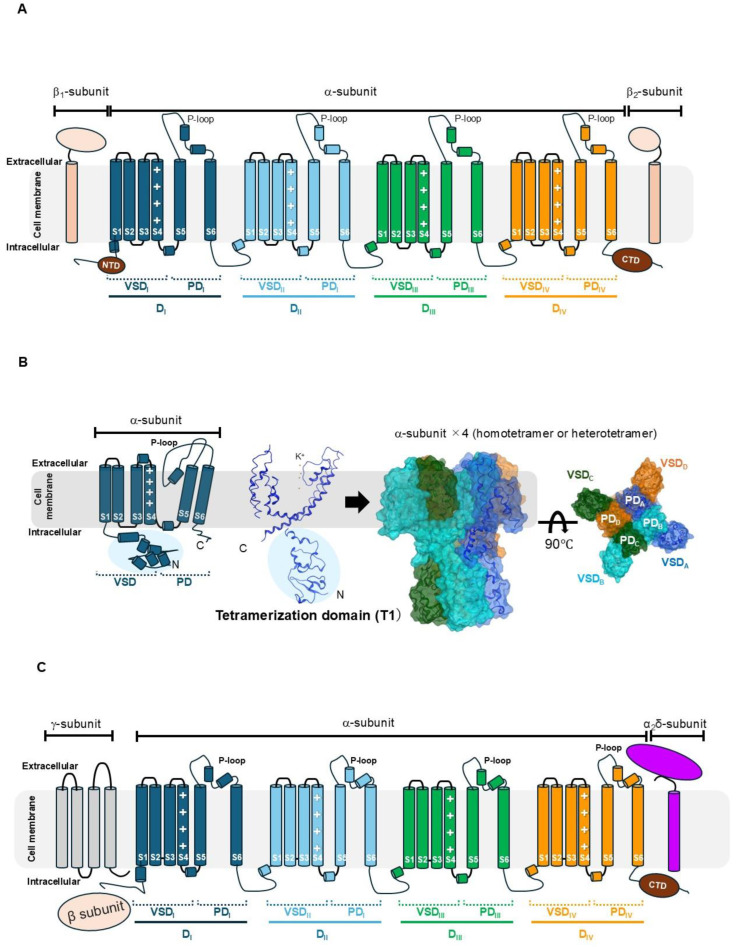
Structural organization of mammalian VGICs (NaV, Kv, and CaV). VGICs adopt a conserved modular scaffold consisting of six transmembrane helices (S1–S6) subdivided into the voltage-sensing domain (VSD; S1–S4), anchored by the positively charged S4 helix, and the pore domain (PD; S5–S6 + P-loop), which hosts the ion-selective filter and permeation pathway. Depolarization-induced motions within the VSD are transmitted allosterically to the PD to open the channel. This shared framework underpins NaV, Kv, and CaV channels alike, whereas subtype-specific architectural features fundamentally shape their physiological functions and pharmacological modulation. (**A**) NaV channels—The α-subunit consists of a single polypeptide containing four homologous domains (DI–DIV), each organized as a VSD–PD module. Inactivation occurs via the intracellular linker between domains III and IV, and auxiliary β subunits (β_1_/β_2_) modulate trafficking and gating. (Model based on human NaV1.7; PDB 7W9L.) (**B**) Kv channels—A functional pore is formed by the tetrameric assembly of four individual α-subunits, each contributing one VSD–PD module. The cytoplasmic N-terminal tetramerization domain organizes subunit assembly, allowing homo- or heterotetramer formation and extensive functional diversity. (Model based on human Kv3.1; PDB 7PQT). (**C**) CaV channels—A single α_1_ subunit contains four homologous VSD–PD modules, similar to NaV channels, but requires three auxiliary subunits (β, α_2_δ, γ) that fine-tune gating, trafficking, and membrane localization. The selectivity filter in the PD coordinates Ca^2+^ to ensure CaV-specific ion permeation. (Model based on human CaV2.3; PDB 7XLQ).

To better understand the toxin binding and modulation mechanisms, it is essential to first outline the structural organization of ion channels along the transmembrane axis, from the extracellular to the intracellular side. As shown in Figure 2, on the extracellular side, the extracellular space provides the initial environment where peptide or protein toxins approach and recognize their targets. Just beneath this lies the outer vestibule, which is a funnel-shaped entryway that guides ions and often serves as a key toxin-binding site [111,112]. Deeper within the pore, the selectivity filter precisely discriminates between different ions (e.g., Na^+^, K^+^, or Ca^2+^) based on size and coordination geometry, thereby defining the channel’s ionic specificity [113,114]. Below the filter, the central cavity (also referred to as the inner vestibule) acts as an aqueous chamber that accommodates hydrated ions before they exit the cytoplasm [115]. At the intracellular end, the intracellular gate controls the ion flow by opening or closing in response to voltage or ligand stimuli, ultimately determining the conductive state of the channel [116]. Finally, the cytoplasmic space interfaces with intracellular signaling molecules and auxiliary subunits that modulate the channel kinetics and regulation [114]. This topological framework provides a basis for interpreting how toxins access and stabilize specific conformational states of the channels. While auxiliary subunits (β, α_2_δ, and γ for CaV; β for Kv; β_1_/β_2_ for NaV) are not discussed in detail in the context of toxin sensitivity in this review, they are included in Figure 2 because they constitute essential components of native VGIC complexes and critically influence channel trafficking, kinetics, and voltage dependence. As recent cryo-EM studies increasingly resolve full channel assemblies rather than isolated α-subunits, future investigations of toxin interactions will likely need to consider the structural and functional contributions of auxiliary subunits. Although current structural data on toxin–auxiliary subunit interfaces remain limited, the inclusion of auxiliary subunits in Figure 2 reflects an emerging trend in the field: toxin binding and conformational stabilization should ultimately be interpreted in the context of the entire channel complex, rather than the α-subunit alone.

### 3.1. Structural Determinants of NaV–Toxin Interactions

NaV channels undergo conformational transitions among closed, activated, and inactivated states in response to changes in the membrane potential. Upon depolarization, channel activation allows rapid Na^+^ influx, initiating the rising phase of the action potential. Shortly thereafter, the channels enter an inactivated state through closure of the inactivation gate, thereby terminating Na^+^ entry and enabling membrane repolarization. Failure or delay of this inactivation process—such as that induced by certain gating-modifier toxins—results in prolonged Na^+^ current and sustained depolarization, leading to neuronal hyperexcitability. The detailed structural topology of NaV channels is shown in Figure 2A; therefore, this subsection focuses on how toxins interact with and stabilize specific conformational states of NaV channels rather than reiterating basic structural elements [117,118,119]. Recent cryo-EM studies have resolved the near-atomic structures of human NaV channels, including NaV1.4, NaV1.5, NaV1.6, and NaV1.7, revealing the arrangement of auxiliary β-subunits that modulate channel trafficking and gating properties [118,120,121,122]. These structures confirm that β1 and β2 subunits associate at extracellular interfaces between VSDs and the pore domain, stabilizing the overall architecture and influencing the channel’s pharmacological and electrophysiological characteristics.

#### 3.1.1. NaV Channel Inhibitors: Pore Blockers and Gating-Modifying Toxins

Inhibitor toxins that target NaV channels act primarily by physically occluding the ion conduction pathway, thereby preventing sodium influx and disrupting normal electrical signaling. These inhibitors typically bind to the extracellular vestibule adjacent to the selectivity filter, with high affinity and isoform-specific selectivity.

ProTx-I exemplifies the gating-modifier class. Cryo-EM structural analysis of the human NaV1.8–ProTx-I complex reveals that the toxin engages the S3–S4 linkers of voltage-sensor domains II and IV, positioning its disulfide-stabilized surface against membrane-exposed loops. This interaction induces a depolarizing shift in the voltage dependence of activation, thereby inhibiting channel opening without occluding the central pore. The high-resolution structure provides direct mechanistic insight into how ProTx-I stabilizes non-conducting VSD conformations and establishes its gating-modifying inhibitory action on NaV1.8 (PDB ID: 9DBN) [123].

HwTx-IV, a gating-modifier peptide, selectively inhibits the human voltage-gated sodium channel NaV1.7 by binding to VSD-II and VSD-IV. Cryo-EM structural analysis revealed that HwTx-IV engages the S3–S4 linkers of both voltage-sensing domains, inducing a depolarizing shift in the voltage dependence of activation. This interaction alters the channel’s gating kinetics, thereby preventing its opening without physically occluding the pore. These findings provide a detailed structural basis for the gating-modifier action of HwTx-IV on NaV1.7 (PDB ID: 7K48) [124].

#### 3.1.2. NaV Channel Activators: Gating-Modifying Peptide Toxins

NaV channel activators increase sodium influx primarily by modifying gating kinetics, either by delaying fast inactivation or by shifting the voltage dependence of activation. Unlike pore-blocking toxins, these activators act allosterically by binding to VSDs and altering channel conformational transitions. However, high-resolution structural complexes of these toxins with NaV channels have not yet been reported.

Herein, we summarize the diverse molecular mechanisms through which toxins modulate NaV channels.

Both peptide and non-peptidic toxins bind with high affinity to specific, functionally critical sites; however, their mechanisms of action differ. Non-peptidic pore blockers, such as TTX (PDB:6A91) and saxitoxin, occlude the selectivity filter, preventing Na^+^ influx with minimal effect on gating [6]. Similarly, pore-blocking peptide toxins, e.g., μ-Conotoxin GIIIA, isolated from the venom of the cone snail *Conus geographus*, inhibit the skeletal muscle NaV1.4 isoform by binding to the extracellular pore vestibule, where it physically obstructs ion permeation [31,32,125]. Mutagenesis and electrophysiological studies have identified critical residues in the outer pore (e.g., Glu403, Lys1237, Asp1241) that interact with μ-GIIIA, supporting its role as a pore-blocking toxin rather than a gating modifier [18,126]. However, the high-resolution structural complexes of toxins with NaV channels have not yet been reported. In contrast, gating-modifying toxins—including batrachotoxin, Hm1a/Hm1b, and scorpion β- and δ-toxins—bind allosterically to voltage-sensing domains or inactivation machinery, altering activation/inactivation kinetics and stabilizing open conformations without directly blocking the pore [127]. This mechanistic dichotomy highlights the diverse molecular strategies that have evolved to regulate the NaV function. Auxiliary β-subunits stabilize the extracellular vestibule and influence access to pharmacologically relevant sites, shaping both gating and ligand recognition. High-resolution human NaV structures have identified conserved receptor sites (1–7) where toxins and therapeutic compounds act [128]. For example, site 1 (S5–S6 loops) is targeted by TTX and STX, while site 3 (S3–S4 linker, domain IV) binds gating-modifier toxins such as δ-atracotoxins and sea anemone toxins, inhibiting fast inactivation [129,130].

### 3.2. Structural Features and Interaction Details of Kv and Toxin

In excitable cells, Kv channels play a central role in membrane repolarization following depolarization. Inhibition of Kv channels reduces K^+^ efflux, delays repolarization, and prolongs action potentials, thereby increasing cellular excitability. Conversely, channel activation enhances K^+^ efflux, accelerates repolarization, and shortens action-potential duration, resulting in reduced excitability. Kv channels are tetrameric voltage-gated K^+^ channels that regulate electrical excitability in neurons, muscle cells, and other excitable tissues. Functional channels assemble as homotetramers or heterotetramers depending on the subtype and physiological context, and this diversity contributes to isoform-specific modulation.

The structural architecture of Kv channels is depicted in Figure 2B based on human Kv3.1 [131]. Kv channels differ fundamentally from NaV and CaV channels in that tetramerization is mediated by a cytoplasmic N-terminal tetramerization domain, rather than being encoded within a single polypeptide chain. This structural organization provides the basis for the formation of distinct α-subunit combinations and underlies subtype-dependent gating and pharmacological properties. Kv channels can also associate with auxiliary Kvβ subunits, which regulate α-subunit expression, membrane trafficking, and gating properties and are most prominently observed in Kv1 and Kv4 subfamilies, such as Kv1 and Kv4 [132].

Venom-derived peptide toxins have evolved to modulate Kv channels with remarkable specificity and affinity. These toxins can be broadly categorized as (i) pore blockers that sterically occlude the ion-conduction pathway and (ii) gating modifiers that allosterically alter activation or inactivation by interacting with voltage-sensing machinery. A subset of peptides instead function as positive modulators (“openers”), stabilizing the open conformation and enhancing K^+^ efflux [133,134]. Structural and biophysical studies have shown that most Kv toxins bind to the extracellular vestibule or the VSD, utilizing conserved toxin–channel interfaces to achieve isoform-specific modulation. Understanding these molecular interactions provides crucial insights into Kv-channel gating and supports the rational design of selective Kv modulators for therapeutic applications [4,135].

#### 3.2.1. Kv Channel Inhibitors: Pore Blockers and Gating-Modifying Toxins

Kv channel antagonist toxins primarily inhibit ion conduction either by physically blocking the channel pore or by modulating the gating properties. These toxins impair repolarization by obstructing potassium efflux, leading to prolonged depolarization and enhanced cellular excitability. They typically bind competitively to the extracellular vestibule near the selectivity filter, act independently of the gating state of the channel, and confer isoform-specific effects depending on their binding interface.

AgTx2, a 38-residue peptide found in the venom of the scorpion *Leiurus quinquestriatus*, acts as a potent pore blocker of Shaker-type Kv channels, including Kv1.1 and Kv1.3. High-speed atomic force microscopy and mutational analyses have revealed that AgTx2 binds to the extracellular vestibule of the channel, positioning key residues over the entrance of the selectivity filter. This interaction sterically occludes K^+^ conduction without altering the intrinsic gating of the channel. Real-time imaging has further demonstrated that binding follows an induced-fit mechanism, in which the channel undergoes subtle conformational adjustments to accommodate the toxin, thereby accelerating the association rate. Although the structure of AgTx2 alone has been resolved using NMR (PDB ID: 1AGT), the high-resolution structure of the AgTx2–Kv channel complex remains to be determined or deposited in the PDB. These studies collectively provide a detailed mechanistic understanding of the AgTx2-mediated pore blockade in Kv channels [136].

#### 3.2.2. Kv Channel Activators: Gating-Modifying Peptide Toxins

Kv channel agonist toxins enhance channel activity by modulating voltage-dependent gating, typically by interacting with the VSD. These peptides shift the activation thresholds to more hyperpolarized potentials or alter the gating kinetics to increase the probability of channel opening. Gating-modifier antagonists do not occlude the pore directly, but bind allosterically to the VSD, altering the voltage dependence of activation or inactivation to inhibit channel function. This mechanism provides a more subtle and tunable form of channel inhibition.

Although several gating-modifying toxins such as SGTx1 and VSTx1 have been shown to modulate Kv channel gating and enhance channel activity through VSD interactions, no high-resolution cryo-EM structures of Kv channels in complex with these activator toxins have yet been reported [137]. Functional assays and fluorescence mapping suggest that these toxins stabilize specific conformational states of the VSD, thereby shifting the activation voltage and modulating the open probability. However, the precise molecular interactions at the protein–lipid interface remain unresolved. This gap highlights the current limitations in structural understanding and underscores the need for future cryo-EM studies to elucidate detailed binding modes and mechanisms by which gating-modifying peptides enhance Kv channel function [137].

The distinct binding modes and mechanisms of action of Kv channel-targeting inhibitor and activator toxins underscore the diverse strategies that evolved using venom peptides to modulate channel function and provide valuable templates for pharmacological interventions. Inhibitor toxins primarily act as direct physical blockers of the ion conduction pathway, with most peptides, including Charybdotoxin, AgTx2, and Maurotoxin, binding to the extracellular vestibule near the selectivity filter and occluding the pore by inserting critical residues, often lysines, into the filter entrance, preventing ion permeation independent of the gating state and, due to their high affinity, typically resulting in prolonged or nearly irreversible channel blockade. In contrast, activator toxins predominantly target the voltage-sensing domain, especially the S3–S4 paddle motif, modulating voltage-sensor movements allosterically rather than physically blocking the pore. For example, SGTx1, and VSTx1 shift the voltage dependence of activation to more hyperpolarized potentials and alter gating kinetics, thereby facilitating channel opening by stabilizing specific conformational states rather than occluding the ion conduction pathway.

### 3.3. Structural Features and Interaction Details of CaV and Toxin

CaV channels play fundamental roles in coupling membrane depolarization to neurotransmitter release, excitation–contraction coupling in muscles, gene transcription, and hormone secretion. These channels comprise a central pore-forming α_1_ subunit together with auxiliary β, α_2_δ, and γ subunits that fine-tune channel expression and gating properties (Figure 2C) [138]. Within the pore, a conserved EEEE or EEDD selectivity-filter motif enables high-affinity Ca^2+^ coordination and selective divalent-cation permeation while excluding monovalent Na^+^ ions. This Ca^2+^-selective filter contrasts with the DEKA motif of NaV channels and the TVGYG motif of Kv channels [139,140]. 

Natural peptide toxins have evolved as potent and subtype-selective modulators of CaV channels. For example, ω-conotoxin MVIIA (ziconotide) blocks CaV2.2 (N-type) channels by binding to the extracellular vestibule, thereby suppressing presynaptic Ca^2+^ influx and neurotransmitter release [141]. Similarly, ω-agatoxin IVA selectively inhibits Caᵥ2.1 (P/Q-type) channels, while SNX-482 targets Caᵥ2.3 (R-type) channels, both acting as pore-blocking antagonists that prevent Ca^2+^ entry [142]. All structurally characterized Caᵥ–toxin complexes represent inhibitory interactions, and no activator-type peptide toxin has been structurally resolved. Although compounds such as maitotoxin and kurtoxin have been reported to alter CaV channel gating, their effects remain incompletely defined and are likely pleiotropic. 

Functionally, CaV channels serve as critical triggers of intracellular calcium signaling. In excitable cells, inhibition of CaV channels reduces Ca^2+^ influx, leading to decreased neurotransmitter or hormone release and attenuated muscle contraction, ultimately lowering cellular excitability. Conversely, activation or prolonged opening of CaV channels enhances Ca^2+^ entry, which can increase neurotransmission and contractility but may also induce calcium overload and excitotoxicity if unregulated. Thus, CaV channel modulators profoundly influence excitability and synaptic function, making them important pharmacological targets in pain, epilepsy, and cardiovascular disorders [143].

ω-Conotoxin MVIIA (Ziconotide): MVIIA is a 25-residue conopeptide from *Conus magus* that selectively blocks N-type CaV2.2 channels by lodging in the extracellular pore vestibule and occluding ion permeation. The high-resolution cryo-EM structure of human CaV2.2 bound to ziconotide (PDB ID 7MIX) directly shows the toxin in the outer vestibule contacting the selectivity filter and extracellular loops. These structural data underpin its subtype selectivity and clinical analgesic action [144], showing that the toxin lodges in the outer pore vestibule, contacting the selectivity filter and extracellular loops of domains II–IV, with critical interactions between Arg10 and Tyr13 of the toxin and acidic residues, such as Asp664 of the channel. These structures highlight the molecular basis for the exquisite subtype selectivity of MVIIA.

ω-Conotoxin MVIIC: another conotoxin, blocks both CaV2.1 (P/Q-type) and CaV2.2 (N-type) channels with high affinity. Structural work has captured the CaV2.1–MVIIC complex [145], showing the toxin engaging the outer pore and extracellular loops of domains I–III, as a pore-occluding inhibitor (CaV2.1/CaV2.2). Toxin residues such as Lys2, Thr11, and Tyr13 form stabilizing interactions with the P-loop and extracellular loop residues of CaV2.1, providing structural confirmation of the mutagenesis data and clarifying the determinants of subtype-specific affinity (PDB ID: 8X91) [146].

Calciseptine: a peptide toxin from *Dendroaspis* (mamba) venom, selectively inhibits L-type CaV1.2 channels. Cryo-EM structures of the human CaV1.2 channel in an inactivated conformation complexed with calciseptine reveal that the toxin binds at the extracellular “shoulder” region, contacting the P2 helices and extracellular loops between Domains III and IV (PDB ID: 8WE7) [147]. This interaction stabilizes the inactivated state rather than physically plugging the central pore. Calciseptine is therefore classified as a pore-proximal inhibitor that stabilizes the inactivated conformation rather than as a gating-modifier toxin that acts through direct interaction with VSDs.

ω-Agatoxin IVA: isolated from the spider (*Agelenopsis aperta*), potently inhibits CaV2.1 (P/Q-type) channels and thereby blocks presynaptic Ca^2+^ influx at central synapses. High-resolution cryo-EM analysis of the human CaV2.1–agatoxin IVA complex (PDB ID: 8X93) [146] revealed binding near the S3–S4 loop of domain IV, consistent with prior mutagenesis mapping. The toxin interacts with the extracellular loops of domain IV via its positively charged arginine-rich patch and C-terminal tail, acting as a gating modifier that hinders voltage-sensor activation and stabilizes the closed state of the channel.

## 4. Disease-Related Ion Channels and Drug Discovery Based on Toxin Studies

Venom-derived peptides are valuable sources of pharmacologically active molecules due to their high affinity and selectivity for ion channels and receptors. Many of these peptides possess disulfide-rich scaffolds, most notably the ICK motif, which confers exceptional thermal stability, proteolytic resistance, and conformational rigidity, enhancing their drug-like properties [26]. To provide a concise overview of the relationships among ion channels, representative toxins, mechanisms of action, and disease relevance, a comprehensive summary is presented in Table 1.

A landmark clinical success in toxin-based drug discovery is Ziconotide (Prialt^®^), a synthetic analog of ω-conotoxin MVIIA from the marine cone snail *Conus magus*. Ziconotide selectively inhibits N-type voltage-gated calcium channels (CaV2.2), blocking neurotransmitter release in the dorsal horn of the spinal cord and producing potent analgesia. It was approved by the U.S. Food and Drug Administration in 2004 for the management of severe chronic pain refractory to conventional therapies [148,149].

Another notable example is Dalazatide (ShK-186), a synthetic analog of the sea anemone peptide ShK. Dalazatide acts as a potent and selective Kv1.3 channel blocker, modulating effector memory T cell function, and thus exhibits immunosuppressive potential. In a Phase 1b clinical trial in patients with plaque psoriasis, Dalazatide was generally well tolerated and reduced both skin lesion severity and inflammatory biomarkers [150]. Preclinical and clinical data have indicated that Dalazatide is a milestone in Kv1.3-targeted immunotherapy [151].

Similarly, HsTX1[R14A], a rationally engineered analog of the scorpion toxin HsTX1, selectively inhibits Kv1.3 with sub-nanomolar potency and has demonstrated favorable pharmacokinetic and immunomodulatory properties in preclinical studies, with no clinical trials reported to date [152]. Structurally, ziconotide, Dalazatide, and HsTX1[R14A] share a compact, cysteine-knotted topology stabilized by multiple disulfide bridges, which not only ensures remarkable stability under physiological conditions but also contributes to prolonged bioactivity relative to linear peptides [153].

These peptides target distinct ion channels and thus modulate different pathophysiological processes:

Ziconotide and its analog Leconotide (ω-conotoxin CVID) block CaV2.2 channels, suppressing neurotransmitter release to exert analgesic effects [95,154,155].

Dalazatide and HsTX1[R14A] block Kv1.3 channels, suppressing effector T cell activation, and provide therapeutic potential for autoimmune and inflammatory diseases [156].

To complement these peptide-based strategies, several small-molecule modulators of disease-related ion channels have also been identified through conventional screening approaches. For example, NaV1.1 activators such as AA43279 have shown therapeutic potential for Dravet syndrome by enhancing the excitability of GABAergic interneurons [50,157]. Although the precise structural basis of their interaction with NaV channels remains unresolved [158], these compounds illustrate how small molecules can modulate ion channel activity through mechanisms that are conceptually distinct from peptide toxins. Thus, toxin-derived peptides and small-molecule modulators represent complementary strategies for targeting ion channels implicated in neurological and immunological disorders.

Taken together, these examples highlight how venom-derived peptides, particularly those utilizing cysteine-rich knottin scaffolds, serve as precise molecular probes and promising therapeutic leads for targeting ion channels previously considered challenging to drug. The continued integration of structural biology, rational peptide design, and toxin-inspired pharmacology is expected to expand this emerging frontier of ion channel drug discovery.

## 5. Bioengineering and Synthetic Modifications for Improved Pharmacological Properties

### 5.1. Medical Applications of Chemical Molecule Modulating Ion Channel

Ion channels in the cell membrane regulate the passage of ions across the cell membrane, thereby controlling various physiological processes. Recent advances in the structural analyses of proteins have led to a deeper understanding of the mechanisms by which ion channel activity is regulated. Based on these findings, various methods have been developed to modulate ion channel functions using chemically synthesized small molecules. In this section, we discuss recent studies on the application of chemically synthesized low-molecular-weight compounds for the treatment of blindness-related diseases.

### 5.2. Degenerative Retinal Diseases

Visual function is a vital sensory modality that is essential for the quality of life. Retinitis pigmentosa (RP) and age-related macular degeneration (AMD), caused by retinal degeneration, are incurable forms of blindness with no effective treatment, ultimately resulting in irreversible vision loss [159,160]. RP is a genetic disorder characterized by the progressive degeneration of photoreceptor cells (rod and cone cells). In patients with RP, peripheral vision loss gradually progresses over several decades, eventually leading to complete blindness; however, no effective treatment has been established. AMD is a leading cause of blindness in developed countries and is clinically classified into wet and dry AMD. Wet AMD is characterized by macular damage caused by abnormal neovascularization, for which anti-vascular endothelial growth factor therapy is available. Dry AMD is a disease characterized by localized visual impairment due to the degeneration of photoreceptor cells in the retinal pigment epithelium and macula; however, no effective treatment has been established. Therefore, the development of new treatment strategies for these intractable retinal diseases is urgently needed.

## 6. Therapeutic Strategies for Restoring Vision

### 6.1. Gene Therapy, Stem Cell Therapy, and Visual Prostheses

Various strategies have been investigated to restore visual function in patients with retinal degenerative diseases, including gene therapy, cell-based therapy, and artificial retinal prostheses [161,162]. Gene therapy includes approaches such as replacing disease-causing genes or introducing photosensitive microbial molecules such as channel rhodopsin-2 and halo rhodopsin into retinal ganglion cells (RGCs) to confer light sensitivity [163,164]. However, it is difficult to evaluate the long-term safety and efficacy of using viral vectors for gene delivery. Stem cell therapy aims to reconstruct visual signal transmission pathways within the retina by replacing degenerated retinal cells with stem cells [165]. Although this approach is promising, there are challenges related to ethical and regulatory issues, and safety evaluations [166,167]. Artificial retinas attempt to restore visual function by electrically stimulating the surviving RGCs using multielectrode arrays implanted in the eye, thereby eliciting visual perceptions known as phosphenes [168]. However, the quality of vision achieved with these systems depends heavily on the number and density of stimulating electrodes and remains markedly inferior in resolution compared to natural vision. Efforts have been made to enhance the performance of stimulating electrodes to improve spatial resolution [169,170,171,172]; still, achieving high-density stimulation at the cellular level within the retina remains technically unfeasible. Currently, visual prostheses that stimulate areas other than the retina, such as the optic nerve and visual cortex, are under development [168].

### 6.2. Photopharmacology Using a Chemical Photoswitch

In recent years, increasing attention has been directed toward photopharmacology, a novel therapeutic approach that modulates neuronal excitability using light-responsive compounds that act on ion channels in degenerated retinal cells [161,173]. This strategy employs photoisomerizable small molecules known as chemical photoswitches to reversibly regulate the opening and closing of ion channels. These chemical photoswitches undergo light-induced isomerization, which alters their molecular structure and, consequently, their binding affinity for target ion channels. This allows for precise and reversible control of channel activity. Azobenzene derivatives are commonly used as core photoresponsive structures, offering high spatiotemporal resolution owing to their well-characterized photochemical properties [174]. Unlike gene or cell therapies, photopharmacology does not require genetic manipulation or cell transplantation and enables the rapid, reversible modulation of neural function [161]. Drug administration to the degenerated retina is performed via intravitreal injection, which is a minimally invasive procedure. Moreover, chemical photoswitches are metabolized and cleared from the body, allowing for flexible adjustment or discontinuation of treatment as needed. Given these advantages, photopharmacology using chemical photoswitches has a strong potential as a novel therapeutic strategy for retinal degenerative diseases.

### 6.3. Features of Chemical Photoswitches

**Figure 3:**
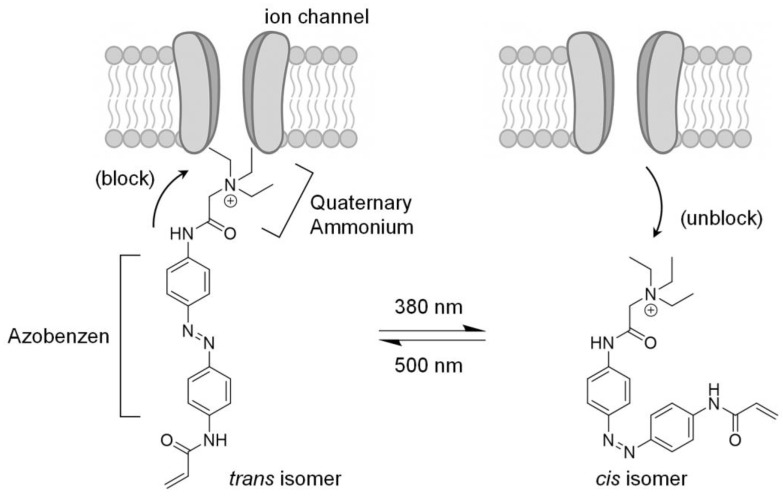
Structural features of AAQ and changes in photoisomerization-dependent affinity for ion channels. AAQ has a photoisomerizable azobenzene core unit and a quaternary ammonium group that exhibits affinity for specific ion channels.

Chemical photoswitches are composed of two key functional components: A ligand moiety that exhibits affinity for the target ion channel, and a central azobenzene core that is photoisomerizable in response to light (Figure 3) [175,176]. To confer ion channel affinity, a quaternary ammonium (QA) group, known to block voltage-gated potassium (Kv) channels, is incorporated into the molecular structure. Upon irradiation at wavelengths near 380 and 500 nm, these molecules exhibit absorption bands in the ultraviolet (UV) region and undergo reversible photoisomerization between the *trans* isomer, which exhibits channel-blocking activity, and the *cis* isomer, which lacks such activity. Upon light stimulation, the azobenzene core undergoes structural conversion from the *trans* isomer to the *cis* isomer, resulting in reduced binding affinity for the target ion channel and subsequent release of the channel blockade. This light-dependent isomerization mechanism enables reversible on/off control of ion channel activity in a temporally and spatially precise manner (Figure 3).

### 6.4. First-Generation Chemical Photoswitch

The first generation of chemical photoswitches is acrylamide azobenzene quaternary ammonium (AAQ) (Figure 3) [175]. AAQ was designed as an open-channel blocker capable of modulating the activity of Kv channels and hyperpolarization-activated cyclic nucleotide-gated channels in a light-dependent manner. AAQ can regulate neuronal excitability upon light stimulation in cultured cells expressing Kv channels as well as in hippocampal neurons from mice [175,177]. However, AAQ requires UV light for photoisomerization, which causes in vivo tissue damage. In addition to the toxicity potential associated with its acrylamide group, AAQ has a short intravitreal half-life of <24 h, which significantly limits its clinical utility. Repeated intravitreal injections every 12–24 h pose a significant burden on both patients and clinicians [162].

### 6.5. Second-Generation Chemical Photoswitches

**Figure 4:**
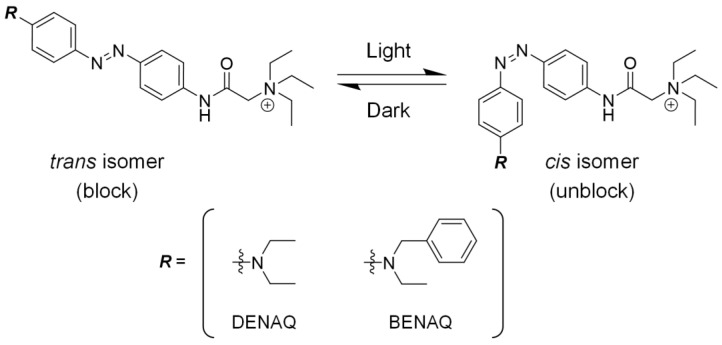
Chemical photoswitches DENAQ and BENAQ. Visible light converts DENAQ and BENAQ from the trans isomer to the cis isomer, and the molecules quickly relax back to the trans isomer in the dark.

To overcome the limitations of AAQ, improved chemical photoswitches, diethyl aminoazobenzene quaternary ammonium (DENAQ) and benzyl ethyl aminoazobenzene quaternary ammonium (BENAQ), were developed (Figure 4) [174]. DENAQ is a derivative in which the acrylamide group of AAQ is replaced by a diethylamino group. It has a “push–pull” azobenzene structure that incorporates both electron-donating and electron-withdrawing substituents. This molecular design significantly red-shifts the absorption spectrum, enabling photoisomerization under visible light rather than under UV irradiation [178].

DENAQ confers light sensitivity to RGCs via hyperpolarization-activated cyclic nucleotide-gated channel photoregulation and induces action potentials in response to light stimulation [179]. In a blind animal model (rd1 mice), a single intravitreal injection of DENAQ enabled the excised retina to maintain photosensitivity for up to seven days post-injection, with no apparent signs of toxicity [179]. However, its relatively short intravitreal half-life of approximately two days remains a limitation for sustained therapeutic efficacy [179].

To address this issue, BENAQ was developed [180]. BENAQ is structurally modified from DENAQ by replacing the ethyl group with a benzyl group, resulting in a significantly extended biological half-life. In rd1 mice, a single intravitreal injection of BENAQ conferred light sensitivity to RGCs for up to one month. Moreover, BENAQ demonstrated 20-fold higher potency than DENAQ, and no toxicity was observed in mice or rabbits, even at doses ten times higher than the dose required for the effect [180]. Recently, the safety and efficacy of BENAQ were confirmed in a clinical trial involving six patients with visual impairments [181]. No severe adverse events, such as intraocular inflammation, were reported, and light-evoked responses were observed. These findings suggest that BENAQ is a promising candidate for the treatment of blindness caused by retinal degeneration and warrants further clinical development.

### 6.6. Acceleration of Drug Development

This section shows that, once bioactive candidate compounds are identified, they can be modified into clinical drugs effectively using established chemical engineering technologies. However, searching for promising lead molecules is currently very time-consuming and costly, which represents a significant bottleneck in drug development. A key strategy to overcome this challenge is to apply expertise from toxin research, given that toxins exhibit remarkably precise physiological effects. Elucidating the specific interactions between toxins and ion channels can significantly improve drug screening efficiency. This approach accelerates the identification of novel bioactive substances and facilitates the development of effective new therapeutics.

## 7. Conclusions and Future Challenges

Small guanidinium toxins, such as tetrodotoxin (TTX) and saxitoxin (STX), have established a canonical framework for pore-occluding inhibition of voltage-gated sodium (NaV) channels. These molecules bind with high affinity to the outer vestibule and interact with acidic residues surrounding the selectivity filter, and effectively occlude ion permeation in a near-stoichiometric manner [111]. Their actions, while highly selective for TTX-sensitive isoforms, exert minimal allosteric influence on gating transitions. Accordingly, TTX and STX have primarily served as structural and functional probes for delineating extracellular pore architecture rather than as modulators of gating kinetics. In contrast, venom-derived peptides exhibit broader mechanistic diversity. Pore-blocking peptides, such as μ-conotoxins at NaV channels, and charybdotoxin and agitoxin at voltage-gated potassium (Kv) channels, act as molecular plugs within the outer vestibule. These peptides often achieve remarkable subtype discrimination by recognizing extended epitope mosaics spanning turret and pore-loop regions [182]. Gating-modifier toxins, on the other hand, engage VSDs to reshape the energy landscape of activation or inactivation. Scorpion α-toxins bias the domain IV VSD (DIV-VSD) to slow inactivation, whereas β-toxins stabilize the domain II/III VSDs (DII/III-VSDs) in pre-activated states, thereby shifting activation thresholds. Tarantula toxins, such as Hm1a, Hm1b, and the hanatoxin family, interact with the S3–S4 loops of VSDs to delay inactivation or stabilize closed states [6,25,183]. Recent cryo-electron microscopy (cryo-EM) structures and comprehensive mutational mapping have visualized discrete binding epitopes, providing a molecular rationale for long-standing electrophysiological observations [124].

Additionally, it is important to recognize that the functional outcomes of toxin–channel interactions are strongly dependent on the cellular and tissue context, as emphasized in the Introduction. Identical ion channel subtypes can produce distinct physiological effects depending on the cell type in which they are expressed. The example of SNX-482 highlights the potential for peptide toxins to act on multiple ion channels. Although widely used as a Cav2.3 inhibitor, SNX-482 also potently inhibits Kv4 family channels, with an IC_50_ lower than for Cav2.3 [82]. This promiscuity underscores the limitations of conventional electrophysiological approaches that often assume a peptide toxin interacts exclusively with a single channel. In practice, most cells express multiple types of ion channels, and the pattern of channel expression can vary between cell types. Consequently, interactions with additional channels may contribute to observed effects, emphasizing the importance of considering potential off-target activities when interpreting experimental results and designing selective channel modulators. For instance, Kv1 channel inhibition may prolong action potentials in cardiac myocytes, yet induce tonic firing in neurons, while even within neuronal populations, the same Kv1 isoform can serve different roles depending on the neuronal subtype. This context-dependent functional diversity is exemplified by peptide toxins such as the spider venom Hm1a, a selective NaV1.1 activator. Hm1a, a spider-venom peptide that demonstrates a strong preference for NaV1.1 over other NaV isoforms, interacts with VSD-IV to suppress inactivation and thereby stabilize the open state of the channel. This mechanism enhances the excitability of GABAergic interneurons, exemplifying how a single toxin can exert therapeutically relevant effects in a cell-type-dependent manner through differential ion channel expression profiles.

This dichotomy between pore occlusion and VSD-targeted allostery represents more than a taxonomic distinction; it defines complementary design paradigms. Small molecules offer advantages in rapid, deep-site occupancy and favorable pharmacokinetics, whereas peptides exploit extensive surface contacts to encode isoform- and state-dependent recognition. Such state-aware modulation preserves physiological excitability while selectively suppressing pathological hyperexcitability. In translational contexts, including pain, epilepsy, cardiac arrhythmia, and retinal neuromodulation, precision therapeutics increasingly rely on this capacity for dynamic and state-specific control rather than simple channel blockade [184,185,186].

Despite these advances, several limitations remain. First, although high-resolution cryo-EM structures of voltage-gated ion channels have proliferated in recent years, structural information on toxin–channel complexes—particularly those involving peptide toxins—remains scarce. This constrains our understanding of precise binding interfaces and the structural basis of gating modulation. Second, gating-modifier toxins cannot be uniformly categorized as “activators” or “inhibitors”. Their ultimate effects on ion flux depend on which VSD is targeted and which conformational state (activated, closed, or inactivated) is stabilized, leading to diverse and sometimes opposing physiological outcomes [7,26,171]. Recognizing these context-dependent effects is essential for interpreting electrophysiological data and designing therapeutically viable modulators.

Future research on toxin-inspired ion channel modulators can be envisioned along several complementary axes. First, isoform- and state-specificity can be refined through epitope engineering, deep mutational scanning, and AI-guided interface design, enabling selective targeting of turret and VSD regions. Second, ensemble-based and dynamics-informed approaches, including molecular dynamics-refined cryo-EM models, can guide stabilization of transition states to bias activation or inactivation. Third, consideration of the cellular, membrane, and extracellular context—including lipid composition and channel heteromerization—is essential, as heteromeric K-channels remain understudied, and tools such as conotoxin κM-RIIIJ are emerging to discriminate heteromeric Kv1 channels [187].

Moreover, it is important to recognize that naturally occurring peptide toxins are often limited in supply and can be challenging to synthesize. A prime example is zetekitoxin AB (ZTX-AB), a saxitoxin analog produced by the critically endangered Panamanian golden frog (*Atelopus zeteki*), of which only approximately 0.5 mg remains worldwide. Despite these limitations, modern analytical and synthetic technologies have enabled detailed structural and functional characterization of such scarce toxins. Yotsu-Yamashita et al. (2004) successfully determined the three-dimensional structure of ZTX-AB using high-resolution NMR and mass spectrometry, providing critical insights into its unique chemical features and potent sodium-channel blocking activity [188]. These advances illustrate how state-of-the-art approaches—including total or semi-synthesis, heterologous expression of biosynthetic pathways, and sensitive analytical methods—can preserve knowledge of rare natural toxins and facilitate the design of selective and state-dependent ion channel modulators.

In addition to natural sources, cutting-edge chemical-genetic tools are expanding the repertoire of selective and state-dependent modulators. Recent preprint data from the DuBois laboratory describe a chemogenetic ligand–receptor pair that enables subtype-selective and reversible inhibition of voltage-gated sodium channels [189]. By pairing a synthetic saxitoxin derivative with engineered pore mutations, the authors achieve approximately 100-fold selectivity for a single channel isoform over wild-type NaV1.1–1.4, 1.6, and 1.7, with rapid and reversible modulation of sodium conductance. This approach exemplifies how modern chemical-genetic technologies can complement natural toxin studies, offering precise and tunable control of ion channel function in both basic and translational research contexts.

Taken together, these examples highlight two complementary strategies for expanding the ion channel modulation toolkit: (i) preservation and structural characterization of scarce natural toxins, and (ii) rational engineering of highly selective and reversible modulators. Both approaches underscore the importance of considering channel subtype diversity, cellular context, and the limitations of assuming single-target specificity when interpreting experimental results or designing therapeutically relevant ion channel modulators.

## Figures and Tables

**Table 1:** Summary of peptide toxins and mechanistically toxin-aligned small-molecule ion channel modulators. While the small molecules listed are not toxin derivatives, their pharmacological actions mimic toxin-mediated modulation of specific channel determinants that were originally identified through toxin research.

Ion Channel	Toxin/Molecule	Mechanism	Disease/Pathophysiological Context
CaV2.2 (N-type)	ω-Conotoxin MVIIA (Ziconotide)	Pore blockade	Severe chronic pain, neuropathic pain
CaV2.2	ω-Conotoxin CVID	Selective CaV2.2 inhibitor	Chronic and neuropathic pain
Kv1.3	ShK/Dalazatide	Kv1.3 pore blocker	Autoimmune diseases (e.g., psoriasis)
Kv1.3	HsTX1[R14A]	Selective Kv1.3 inhibitor	Autoimmune and inflammatory diseases
NaV1.1	Hm1a/Hm1b	NaV1.1 activation	Dravet syndrome
NaV1.1	AA43279	NaV1.1 gating enhancer	Dravet syndrome
NaV1.7	ProTx-II/HwTx-IV	NaV1.7 inhibition	Pain disorders
CaV1.2	Calciseptine	L-type Ca^2+^ inhibition	Hypertension, arrhythmia
CaV2.1	ω-Agatoxin IVA	P/Q-type Ca^2+^ inhibition	Episodic ataxia, FHM
hERG (Kv11.1)	APETx1	hERG gating inhibition	Long QT syndrome
Kv/HCN (RGCs)	AAQ, DENAQ, BENAQ	Photoswitchable block of Kv and HCN channels in retinal ganglion cells	Retinal degenerative diseases

## Data Availability

No new data were created or analyzed in this study.

## Abbreviations

The following abbreviations are used in this manuscript:

VGIC	Voltage-gated ion channels
VSD	Voltage-sensing domain
NaV	Voltage-gated sodium channel
Kv	Voltage-gated potassium channel
CaV	Voltage-gated calcium channel
TTX	Tetrodotoxin
STX	Saxitoxin
NaTx	Sea anemone sodium channel toxins
ASIC	Acid-sensing ion channel
ICK	Inhibitory cystine knot fold
KTx	Potassium channel toxins
BK	Large-conductance Ca^2+^-activated K^+^ channel
cryo-EM	Cryo-electron microscopy
EMDB	Electron Microscopy Data Bank
PDB	Protein Data Bank
3DEM	Three-dimensional electron microscopy
NMR	Nuclear magnetic resonance
AFM	Atomic force microscopy
hERG	Human ether-à-go-go–related gene (Kv11.1) channel
RGC	Retinal ganglion cell
RP	Retinitis pigmentosa
AMD	Age-related macular degeneration
QA	Quaternary ammonium
UV	Ultraviolet
AAQ	Acrylamide azobenzene quaternary ammonium
DENAQ	Diethyl aminoazobenzene quaternary ammonium
BENAQ	Benzyl ethyl aminoazobenzene quaternary ammonium
PIP_2_	Phosphatidylinositol 4,5-bisphosphate
MD	Molecular dynamics
CSαβ	Cystine-stabilized α/β motif
FDA	U.S. Food and Drug Administration
AgTx2	Agitoxin-2
ChTX	Charybdotoxin
ATX-II	Anemonia toxin II
HwTx-IV	Huwentoxin-IV
ProTx	Protoxin
SNX-482	Spider toxin from *Hysterocrates gigas*
ω-Conotoxin GVIA	Omega-conotoxin GVIA
ω-Conotoxin MVIIA	Omega-conotoxin MVIIA (Ziconotide)
ω-Conotoxin MVIIC	Omega-conotoxin MVIIC
ω-Agatoxin IVA	Omega-agatoxin IVA
APETx1	Anthopleurin-like peptide toxin 1
BDS-I	Blood Depressing Substance I
VSTx1	Voltage-Sensor Toxin 1
HaTx1	Hanatoxin
Hm1	Tarantula toxin from *Heteroscodra maculata*

## References

[1] Felipe A., Ferrer-Montiel A. (2023). Membrane Channels in Health and Diseases. Int. J. Mol. Sci..

[2] Harraz O.F., Delpire E. (2024). Recent Insights into Channelopathies. Physiol. Rev..

[3] Peixoto-Neves D., Jaggar J.H. (2024). Physiological Functions and Pathological Involvement of Ion Channel Trafficking in the Vasculature. J. Physiol..

[4] Deuis J.R., Mueller A., Israel M.R., Vetter I. (2017). The Pharmacology of Voltage-Gated Sodium Channel Activators. Neuropharmacology.

[5] Bae C., Anselmi C., Kalia J., Jara-Oseguera A., Schwieters C.D., Krepkiy D., Lee W.C., Kim E.-H., Kim J.I., Faraldo-Gómez J.D. (2016). Structural Insights into the Mechanism of Activation of the TRPV1 Channel by a Membrane-Bound Tarantula Toxin. Elife.

[6] Shen H., Li Z., Jiang Y., Pan X., Wu J., Cristofori-Armstrong B., Smith J.J., Chin Y.K.Y., Lei J., Zhou Q. (2018). Structural Basis for the Modulation of Voltage-Gated Sodium Channels by Animal Toxins. Science.

[7] Krylov N.A., Tabakmakher V.M., Yureva D.A., Vassilevski A.A., Kuzmenkov A.I. (2023). Kalium 3.0 Is a Comprehensive Depository of Natural, Artificial, and Labeled Polypeptides Acting on Potassium Channels. Protein Sci..

[8] Catterall W.A., Cestèle S., Yarov-Yarovoy V., Yu F.H., Konoki K., Scheuer T. (2007). Voltage-Gated Ion Channels and Gating Modifier Toxins. Toxicon.

[9] Kalia J., Milescu M., Salvatierra J., Wagner J., Klint J.K., King G.F., Olivera B.M., Bosmans F. (2015). From Foe to Friend: Using Animal Toxins to Investigate Ion Channel Function. J. Mol. Biol..

[10] Arias H.R. (2006). Marine Toxins Targeting Ion Channels. Mar. Drugs.

[11] Ariens E.J. (1954). Affinity and Intrinsic Activity in the Theory of Competitive Inhibition. I. Problems and Theory. Arch. Int. Pharmacodyn. Ther..

[12] Weir C.J. (2020). Ion Channels, Receptors, Agonists and Antagonists. Anaesth. Intensive Care Med..

[13] Stephenson R.P. (1956). A Modification of Receptor Theory. Br. J. Pharmacol. Chemother..

[14] Black J.W., Leff P. (1983). Operational Models of Pharmacological Agonism. Proc. R. Soc. Lond. B Biol. Sci..

[15] Swartz K.J., MacKinnon R. (1997). Hanatoxin Modifies the Gating of a Voltage-Dependent K^+^ Channel through Multiple Binding Sites. Neuron.

[16] Li-Smerin Y., Swartz K.J. (1998). Gating Modifier Toxins Reveal a Conserved Structural Motif in Voltage-Gated Ca^2+^ and K^+^ Channels. Proc. Natl. Acad. Sci. USA.

[17] Stevens M., Peigneur S., Tytgat J. (2011). Neurotoxins and Their Binding Areas on Voltage-Gated Sodium Channels. Front. Pharmacol..

[18] Catterall W.A. (2012). Voltage-Gated Sodium Channels at 60: Structure, Function and Pathophysiology: Voltage-Gated Sodium Channels. J. Physiol..

[19] Catterall W.A., Perez-Reyes E., Snutch T.P., Striessnig J. (2005). International Union of Pharmacology. XLVIII. Nomenclature and Structure-Function Relationships of Voltage-Gated Calcium Channels. Pharmacol. Rev..

[20] Clare J.J., Tate S.N., Nobbs M., Romanos M.A. (2000). Voltage-Gated Sodium Channels as Therapeutic Targets. Drug Discov. Today.

[21] Hoffman E.P., Lehmann-Horn F., Rüdel R. (1995). Overexcited or Inactive: Ion Channels in Muscle Disease. Cell.

[22] Plummer N.W., Meisler M.H. (1999). Evolution and Diversity of Mammalian Sodium Channel Genes. Genomics.

[23] Meisler M.H., Hill S.F., Yu W. (2021). Sodium Channelopathies in Neurodevelopmental Disorders. Nat. Rev. Neurosci..

[24] Mantegazza M., Cestèle S., Catterall W.A. (2021). Sodium Channelopathies of Skeletal Muscle and Brain. Physiol. Rev..

[25] de Lera Ruiz M., Kraus R.L. (2015). Voltage-Gated Sodium Channels: Structure, Function, Pharmacology, and Clinical Indications. J. Med. Chem..

[26] King G.F. (2011). Venoms as a Platform for Human Drugs: Translating Toxins into Therapeutics. Expert Opin. Biol. Ther..

[27] Lenaeus M.J., Gamal El-Din T.M., Ing C., Ramanadane K., Pomès R., Zheng N., Catterall W.A. (2017). Structures of Closed and Open States of a Voltage-Gated Sodium Channel. Proc. Natl. Acad. Sci. USA.

[28] Gilchrist J., Olivera B.M., Bosmans F. (2014). Animal Toxins Influence Voltage-Gated Sodium Channel Function. Handb. Exp. Pharmacol..

[29] Li R., Yu J., Ye D., Liu S., Zhang H., Lin H., Feng J., Deng K. (2025). Conotoxins: Classification, Prediction, and Future Directions in Bioinformatics. Toxins.

[30] Li R.A., Tomaselli G.F. (2004). Using the Deadly Mu-Conotoxins as Probes of Voltage-Gated Sodium Channels. Toxicon.

[31] Green B.R., Bulaj G., Norton R.S. (2014). Structure and Function of μ-Conotoxins, Peptide-Based Sodium Channel Blockers with Analgesic Activity. Future Med. Chem..

[32] Wilson M.J., Yoshikami D., Azam L., Gajewiak J., Olivera B.M., Bulaj G., Zhang M.-M. (2011). μ-Conotoxins That Differentially Block Sodium Channels NaV1.1 through 1.8 Identify Those Responsible for Action Potentials in Sciatic Nerve. Proc. Natl. Acad. Sci. USA.

[33] Monastyrnaya M.M., Kalina R.S., Kozlovskaya E.P. (2022). The Sea Anemone Neurotoxins Modulating Sodium Channels: An Insight at Structure and Functional Activity after Four Decades of Investigation. Toxins.

[34] Madio B., King G.F., Undheim E.A.B. (2019). Sea Anemone Toxins: A Structural Overview. Mar. Drugs.

[35] Fletcher J.E., Adnet P.J., Reyford H., Wieland S.J., Stewart S.L., Rosenberg H. (1999). ATX II, a Sodium Channel Toxin, Sensitizes Skeletal Muscle to Halothane, Caffeine, and Ryanodine. Anesthesiology.

[36] Lu Y.-Y., Cheng C.-C., Chen Y.-C., Chen S.-A., Chen Y.-J. (2012). ATX-II-Induced Pulmonary Vein Arrhythmogenesis Related to Atrial Fibrillation and Long QT Syndrome. Eur. J. Clin. Investig..

[37] Cestèle S., Qu Y., Rogers J.C., Rochat H., Scheuer T., Catterall W.A. (1998). Voltage Sensor-Trapping: Enhanced Activation of Sodium Channels by Beta-Scorpion Toxin Bound to the S3-S4 Loop in Domain II. Neuron.

[38] Campos F.V., Coronas F.I.V., Beirão P.S.L. (2004). Voltage-Dependent Displacement of the Scorpion Toxin Ts3 from Sodium Channels and Its Implication on the Control of Inactivation: Scorpion Toxin and Sodium Channel Inactivation. Br. J. Pharmacol..

[39] Jover E., Martin-Moutot N., Couraud F., Rochat H. (1978). Scorpion Toxin: Specific Binding to Rat Synaptosomes. Biochem. Biophys. Res. Commun..

[40] Tejedor F.J., Catterall W.A. (1988). Site of Covalent Attachment of Alpha-Scorpion Toxin Derivatives in Domain I of the Sodium Channel Alpha Subunit. Proc. Natl. Acad. Sci. USA.

[41] Rogers J.C., Qu Y., Tanada T.N., Scheuer T., Catterall W.A. (1996). Molecular Determinants of High Affinity Binding of Alpha-Scorpion Toxin and Sea Anemone Toxin in the S3-S4 Extracellular Loop in Domain IV of the Na+ Channel Alpha Subunit. J. Biol. Chem..

[42] Leipold E., Hansel A., Borges A., Heinemann S.H. (2006). Subtype Specificity of Scorpion Beta-Toxin Tz1 Interaction with Voltage-Gated Sodium Channels Is Determined by the Pore Loop of Domain 3. Mol. Pharmacol..

[43] Klint J.K., Senff S., Rupasinghe D.B., Er S.Y., Herzig V., Nicholson G.M., King G.F. (2012). Spider-Venom Peptides That Target Voltage-Gated Sodium Channels: Pharmacological Tools and Potential Therapeutic Leads. Toxicon.

[44] Bosmans F., Swartz K.J. (2010). Targeting Voltage Sensors in Sodium Channels with Spider Toxins. Trends Pharmacol. Sci..

[45] Cardoso F.C., Lewis R.J. (2019). Structure-Function and Therapeutic Potential of Spider Venom-Derived Cysteine Knot Peptides Targeting Sodium Channels. Front. Pharmacol..

[46] Yuan F.-C., Sun F.-D., Zhang L., Huang B., An H.-L., Rong M.-Q., Du C.-W. (2022). General Mechanism of Spider Toxin Family I Acting on Sodium Channel Nav1.7. Zool. Res..

[47] Nicholson G.M., Walsh R., Little M.J., Tyler M.I. (1998). Characterisation of the Effects of Robustoxin, the Lethal Neurotoxin from the Sydney Funnel-Web Spider Atrax Robustus, on Sodium Channel Activation and Inactivation. Pflugers Arch..

[48] Alewood D., Birinyi-Strachan L.C., Pallaghy P.K., Norton R.S., Nicholson G.M., Alewood P.F. (2003). Synthesis and Characterization of Delta-Atracotoxin-Ar1a, the Lethal Neurotoxin from Venom of the Sydney Funnel-Web Spider (*Atrax Robustus*). Biochemistry.

[49] Ulbricht W. (2005). Sodium Channel Inactivation: Molecular Determinants and Modulation. Physiol. Rev..

[50] Chow C.Y., Chin Y.K.Y., Ma L., Undheim E.A.B., Herzig V., King G.F. (2020). A Selective NaV1.1 Activator with Potential for Treatment of Dravet Syndrome Epilepsy. Biochem. Pharmacol..

[51] Middleton R.E., Warren V.A., Kraus R.L., Hwang J.C., Liu C.J., Dai G., Brochu R.M., Kohler M.G., Gao Y.-D., Garsky V.M. (2002). Two Tarantula Peptides Inhibit Activation of Multiple Sodium Channels. Biochemistry.

[52] Lopez L., Montnach J., Oliveira-Mendes B., Khakh K., Thomas B., Lin S., Caumes C., Wesolowski S., Nicolas S., Servent D. (2021). Synthetic Analogues of Huwentoxin-IV Spider Peptide with Altered Human NaV1.7/NaV1.6 Selectivity Ratios. Front. Cell Dev. Biol..

[53] Jacobsson E., Andersson H.S., Strand M., Peigneur S., Eriksson C., Lodén H., Shariatgorji M., Andrén P.E., Lebbe E.K.M., Rosengren K.J. (2018). Peptide Ion Channel Toxins from the Bootlace Worm, the Longest Animal on Earth. Sci. Rep..

[54] Jacobsson E., Peigneur S., Andersson H.S., Laborde Q., Strand M., Tytgat J., Göransson U. (2021). Functional Characterization of the Nemertide α Family of Peptide Toxins. J. Nat. Prod..

[55] Allen N.M., Weckhuysen S., Gorman K., King M.D., Lerche H. (2020). Genetic Potassium Channel-Associated Epilepsies: Clinical Review of the Kv Family. Eur. J. Paediatr. Neurol..

[56] Zheng Y., Chen J. (2024). Voltage-Gated Potassium Channels and Genetic Epilepsy. Front. Neurol..

[57] Faulkner I.E., Pajak R.Z., Harte M.K., Glazier J.D., Hager R. (2024). Voltage-Gated Potassium Channels as a Potential Therapeutic Target for the Treatment of Neurological and Psychiatric Disorders. Front. Cell. Neurosci..

[58] Finol-Urdaneta R.K., Belovanovic A., Micic-Vicovac M., Kinsella G.K., McArthur J.R., Al-Sabi A. (2020). Marine Toxins Targeting Kv1 Channels: Pharmacological Tools and Therapeutic Scaffolds. Mar. Drugs.

[59] Norton R.S., Chandy K.G. (2017). Venom-Derived Peptide Inhibitors of Voltage-Gated Potassium Channels. Neuropharmacology.

[60] Mouhat S., Andreotti N., Jouirou B., Sabatier J.-M. (2008). Animal Toxins Acting on Voltage-Gated Potassium Channels. Curr. Pharm. Des..

[61] Wulff H., Castle N.A., Pardo L.A. (2009). Voltage-Gated Potassium Channels as Therapeutic Targets. Nat. Rev. Drug Discov..

[62] Shon K.J., Stocker M., Terlau H., Stühmer W., Jacobsen R., Walker C., Grilley M., Watkins M., Hillyard D.R., Gray W.R. (1998). Kappa-Conotoxin PVIIA Is a Peptide Inhibiting the Shaker K^+^ Channel. J. Biol. Chem..

[63] Naranjo D., Díaz-Franulic I. (2020). Binding of κ-Conotoxin-PVIIA to Open and Closed Shaker K-Channels Are Differentially Affected by the Ionic Strength. Mar. Drugs.

[64] Mendes L.C., Viana G.M.M., Nencioni A.L.A., Pimenta D.C., Beraldo-Neto E. (2023). Scorpion Peptides and Ion Channels: An Insightful Review of Mechanisms and Drug Development. Toxins.

[65] Cerni F.A., Pucca M.B., Peigneur S., Cremonez C.M., Bordon K.C.F., Tytgat J., Arantes E.C. (2014). Electrophysiological Characterization of Ts6 and Ts7, K^+^ Channel Toxins Isolated through an Improved Tityus Serrulatus Venom Purification Procedure. Toxins.

[66] de Oliveira I.S., Alano-da-Silva N.M., Ferreira I.G., Cerni F.A., Sachett J.d.A.G., Monteiro W.M., Pucca M.B., Arantes E.C. (2024). Understanding the Complexity of Tityus Serrulatus Venom: A Focus on High Molecular Weight Components. J. Venom. Anim. Toxins Incl. Trop. Dis..

[67] Pucca M.B., Bertolini T.B., Cerni F.A., Bordon K.C.F., Peigneur S., Tytgat J., Bonato V.L., Arantes E.C. (2016). Immunosuppressive Evidence of Tityus Serrulatus Toxins Ts6 and Ts15: Insights of a Novel K(+) Channel Pattern in T Cells. Immunology.

[68] Garcia M.L., Garcia-Calvo M., Hidalgo P., Lee A., MacKinnon R. (1994). Purification and Characterization of Three Inhibitors of Voltage-Dependent K^+^ Channels from Leiurus Quinquestriatus Var. Hebraeus Venom. Biochemistry.

[69] Eriksson M.A.L., Roux B. (2002). Modeling the Structure of Agitoxin in Complex with the Shaker K^+^ Channel: A Computational Approach Based on Experimental Distance Restraints Extracted from Thermodynamic Mutant Cycles. Biophys. J..

[70] MacKinnon R. (1991). Determination of the Subunit Stoichiometry of a Voltage-Activated Potassium Channel. Nature.

[71] Park C.S., Miller C. (1992). Interaction of Charybdotoxin with Permeant Ions inside the Pore of a K^+^ Channel. Neuron.

[72] Chen R., Chung S.-H. (2012). Structural Basis of the Selective Block of Kv1.2 by Maurotoxin from Computer Simulations. PLoS ONE.

[73] Blanc E., Sabatier J.M., Kharrat R., Meunier S., El Ayeb M., Van Rietschoten J., Darbon H. (1997). Solution Structure of Maurotoxin, a Scorpion Toxin from Scorpio Maurus, with High Affinity for Voltage-gated Potassium Channels. Proteins.

[74] Benishin C.G., Sorensen R.G., Brown W.E., Krueger B.K., Blaustein M.P. (1988). Four Polypeptide Components of Green Mamba Venom Selectively Block Certain Potassium Channels in Rat Brain Synaptosomes. Mol. Pharmacol..

[75] Harvey A.L. (2001). Twenty Years of Dendrotoxins. Toxicon.

[76] Imredy J.P., MacKinnon R. (2000). Energetic and Structural Interactions between Delta-Dendrotoxin and a Voltage-Gated Potassium Channel. J. Mol. Biol..

[77] Ruta V., Jiang Y., Lee A., Chen J., MacKinnon R. (2003). Functional Analysis of an Archaebacterial Voltage-Dependent K^+^ Channel. Nature.

[78] Phillips L.R., Milescu M., Li-Smerin Y., Mindell J.A., Kim J.I., Swartz K.J. (2005). Voltage-Sensor Activation with a Tarantula Toxin as Cargo. Nature.

[79] Lee C.W., Kim S., Roh S.H., Endoh H., Kodera Y., Maeda T., Kohno T., Wang J.M., Swartz K.J., Kim J.I. (2004). Solution Structure and Functional Characterization of SGTx1, a Modifier of Kv2.1 Channel Gating. Biochemistry.

[80] Jung H.J., Lee J.Y., Kim S.H., Eu Y.-J., Shin S.Y., Milescu M., Swartz K.J., Kim J.I. (2005). Solution Structure and Lipid Membrane Partitioning of VSTx1, an Inhibitor of the KvAP Potassium Channel. Biochemistry.

[81] Schmidt D., MacKinnon R. (2008). Voltage-Dependent K^+^ Channel Gating and Voltage Sensor Toxin Sensitivity Depend on the Mechanical State of the Lipid Membrane. Proc. Natl. Acad. Sci. USA.

[82] Kimm T., Bean B.P. (2014). Inhibition of A-Type Potassium Current by the Peptide Toxin SNX-482. J. Neurosci..

[83] Diochot S., Loret E., Bruhn T., Béress L., Lazdunski M. (2003). APETx1, a New Toxin from the Sea Anemone Anthopleura Elegantissima, Blocks Voltage-Gated Human Ether-a-Go-Go-Related Gene Potassium Channels. Mol. Pharmacol..

[84] Zhang M., Liu J., Tseng G.-N. (2004). Gating Charges in the Activation and Inactivation Processes of the HERG Channel. J. Gen. Physiol..

[85] Chagot B., Diochot S., Pimentel C., Lazdunski M., Darbon H. (2005). Solution Structure of APETx1 from the Sea Anemone Anthopleura Elegantissima: A New Fold for an HERG Toxin. Proteins.

[86] Matsumura K., Shimomura T., Kubo Y., Oka T., Kobayashi N., Imai S., Yanase N., Akimoto M., Fukuda M., Yokogawa M. (2021). Mechanism of HERG Inhibition by Gating-Modifier Toxin, APETx1, Deduced by Functional Characterization. BMC Mol. Cell Biol..

[87] Diochot S., Schweitz H., Béress L., Lazdunski M. (1998). Sea Anemone Peptides with a Specific Blocking Activity against the Fast Inactivating Potassium Channel Kv3.4. J. Biol. Chem..

[88] Yeung S.Y.M., Thompson D., Wang Z., Fedida D., Robertson B. (2005). Modulation of Kv3 Subfamily Potassium Currents by the Sea Anemone Toxin BDS: Significance for CNS and Biophysical Studies. J. Neurosci..

[89] Rivera-Torres I.O., Jin T.B., Cadene M., Chait B.T., Poget S.F. (2016). Discovery and Characterisation of a Novel Toxin from Dendroaspis Angusticeps, Named Tx7335, That Activates the Potassium Channel KcsA. Sci. Rep..

[90] Helbig K.L., Lauerer R.J., Bahr J.C., Souza I.A., Myers C.T., Uysal B., Schwarz N., Gandini M.A., Huang S., Keren B. (2018). De Novo Pathogenic Variants in CACNA1E Cause Developmental and Epileptic Encephalopathy with Contractures, Macrocephaly, and Dyskinesias. Am. J. Hum. Genet..

[91] Ophoff R.A., Terwindt G.M., Vergouwe M.N., van Eijk R., Oefner P.J., Hoffman S.M., Lamerdin J.E., Mohrenweiser H.W., Bulman D.E., Ferrari M. (1996). Familial Hemiplegic Migraine and Episodic Ataxia Type-2 Are Caused by Mutations in the Ca^2+^ Channel Gene CACNL1A4. Cell.

[92] Splawski I., Timothy K.W., Decher N., Kumar P., Sachse F.B., Beggs A.H., Sanguinetti M.C., Keating M.T. (2005). Severe Arrhythmia Disorder Caused by Cardiac L-Type Calcium Channel Mutations. Proc. Natl. Acad. Sci. USA.

[93] Jiang C., Zhang Y. (2023). Current Updates on Arrhythmia within Timothy Syndrome: Genetics, Mechanisms and Therapeutics. Expert Rev. Mol. Med..

[94] Olivera B.M., Miljanich G.P., Ramachandran J., Adams M.E. (1994). Calcium Channel Diversity and Neurotransmitter Release: The Omega-Conotoxins and Omega-Agatoxins. Annu. Rev. Biochem..

[95] Miljanich G.P. (2004). Ziconotide: Neuronal Calcium Channel Blocker for Treating Severe Chronic Pain. Curr. Med. Chem..

[96] Pinheiro-Junior E.L., Kalina R., Gladkikh I., Leychenko E., Tytgat J., Peigneur S. (2022). A Tale of Toxin Promiscuity: The Versatile Pharmacological Effects of Hcr 1b-2 Sea Anemone Peptide on Voltage-Gated Ion Channels. Mar. Drugs.

[97] Ferron L., Zamponi G.W. (2024). A Tale of Two Calcium Channels: Structural Pharmacology of Cav2.1 and Cav3.2. Cell Res..

[98] Lee C.W., Bae C., Lee J., Ryu J.H., Kim H.H., Kohno T., Swartz K.J., Kim J.I. (2012). Solution Structure of Kurtoxin: A Gating Modifier Selective for Cav3 Voltage-Gated Ca(2+) Channels. Biochemistry.

[99] Sidach S.S., Mintz I.M. (2002). Kurtoxin, a Gating Modifier of Neuronal High- and Low-Threshold ca Channels. J. Neurosci..

[100] Chuang R.S., Jaffe H., Cribbs L., Perez-Reyes E., Swartz K.J. (1998). Inhibition of T-Type Voltage-Gated Calcium Channels by a New Scorpion Toxin. Nat. Neurosci..

[101] Lampe R.A., Defeo P.A., Davison M.D., Young J., Herman J.L., Spreen R.C., Horn M.B., Mangano T.J., Keith R.A. (1993). Isolation and Pharmacological Characterization of Omega-Grammotoxin SIA, a Novel Peptide Inhibitor of Neuronal Voltage-Sensitive Calcium Channel Responses. Mol. Pharmacol..

[102] McDonough S.I., Lampe R.A., Keith R.A., Bean B.P. (1997). Voltage-Dependent Inhibition of N- and P-Type Calcium Channels by the Peptide Toxin Omega-Grammotoxin-SIA. Mol. Pharmacol..

[103] Takeuchi K., Park E., Lee C., Kim J., Takahashi H., Swartz K., Shimada I. (2002). Solution Structure of Omega-Grammotoxin SIA, a Gating Modifier of P/Q and N-Type Ca(2+) Channel. J. Mol. Biol..

[104] Bourinet E., Stotz S.C., Spaetgens R.L., Dayanithi G., Lemos J., Nargeot J., Zamponi G.W. (2001). Interaction of SNX482 with Domains III and IV Inhibits Activation Gating of Alpha(1E) (Ca(V)2.3) Calcium Channels. Biophys. J..

[105] Newcomb R., Szoke B., Palma A., Wang G., Chen X.-h., Hopkins W., Cong R., Miller J., Urge L., Tarczy-Hornoch K. (1998). Selective Peptide Antagonist of the Class E Calcium Channel from the Venom of the Tarantula Hysterocrates Gigas. Biochemistry.

[106] Priest B.T., Blumenthal K.M., Smith J.J., Warren V.A., Smith M.M. (2007). ProTx-I and ProTx-II: Gating Modifiers of Voltage-Gated Sodium Channels. Toxicon.

[107] Schmalhofer W.A., Calhoun J., Burrows R., Bailey T., Kohler M.G., Weinglass A.B., Kaczorowski G.J., Garcia M.L., Koltzenburg M., Priest B.T. (2008). ProTx-II, a Selective Inhibitor of NaV1.7 Sodium Channels, Blocks Action Potential Propagation in Nociceptors. Mol. Pharmacol..

[108] Bladen C., Hamid J., Souza I.A., Zamponi G.W. (2014). Block of T-Type Calcium Channels by Protoxins I and II. Mol. Brain.

[109] Edgerton G.B., Blumenthal K.M., Hanck D.A. (2008). Evidence for Multiple Effects of ProTxII on Activation Gating in Na(V)1.5. Toxicon.

[110] Shen H., Liu D., Wu K., Lei J., Yan N. (2019). Structures of Human Nav1.7 Channel in Complex with Auxiliary Subunits and Animal Toxins. Science.

[111] Payandeh J., Scheuer T., Zheng N., Catterall W.A. (2011). The Crystal Structure of a Voltage-Gated Sodium Channel. Nature.

[112] Shen H., Zhou Q., Pan X., Li Z., Wu J., Yan N. (2017). Structure of a Eukaryotic Voltage-Gated Sodium Channel at near-Atomic Resolution. Science.

[113] Doyle D.A., Morais Cabral J., Pfuetzner R.A., Kuo A., Gulbis J.M., Cohen S.L., Chait B.T., MacKinnon R. (1998). The Structure of the Potassium Channel: Molecular Basis of K^+^ Conduction and Selectivity. Science.

[114] Wu J., Yan Z., Li Z., Yan C., Lu S., Dong M., Yan N. (2015). Structure of the Voltage-Gated Calcium Channel Cav1.1 Complex. Science.

[115] Xu H., Li T., Rohou A., Arthur C.P., Tzakoniati F., Wong E., Estevez A., Kugel C., Franke Y., Chen J. (2019). Structural Basis of Nav1.7 Inhibition by a Gating-Modifier Spider Toxin. Cell.

[116] Sun J., MacKinnon R. (2020). Structural Basis of Human KCNQ1 Modulation and Gating. Cell.

[117] Yan Z., Zhou Q., Wang L., Wu J., Zhao Y., Huang G., Peng W., Shen H., Lei J., Yan N. (2017). Structure of the Nav1.4-Β1 Complex from Electric Eel. Cell.

[118] Pan X., Li Z., Zhou Q., Shen H., Wu K., Huang X., Chen J., Zhang J., Zhu X., Lei J. (2018). Structure of the Human Voltage-Gated Sodium Channel Nav1.4 in Complex with Β1. Science.

[119] Jiang D., Zhang J., Xia Z. (2022). Structural Advances in Voltage-Gated Sodium Channels. Front. Pharmacol..

[120] Jiang D., Shi H., Tonggu L., Gamal El-Din T.M., Lenaeus M.J., Zhao Y., Yoshioka C., Zheng N., Catterall W.A. (2020). Structure of the Cardiac Sodium Channel. Cell.

[121] Huang G., Liu D., Wang W., Wu Q., Chen J., Pan X., Shen H., Yan N. (2022). High-Resolution Structures of Human Nav1.7 Reveal Gating Modulation through α-π Helical Transition of S6IV. Cell Rep..

[122] Fan X., Huang J., Jin X., Yan N. (2023). Cryo-EM Structure of Human Voltage-Gated Sodium Channel Nav1.6. Proc. Natl. Acad. Sci. USA.

[123] Neumann B., McCarthy S., Gonen S. (2025). Structural Basis of Inhibition of Human NaV1.8 by the Tarantula Venom Peptide Protoxin-I. Nat. Commun..

[124] Wisedchaisri G., Tonggu L., Gamal El-Din T.M., McCord E., Zheng N., Catterall W.A. (2021). Structural Basis for High-Affinity Trapping of the NaV1.7 Channel in Its Resting State by Tarantula Toxin. Mol. Cell.

[125] Mahdavi S., Kuyucak S. (2014). Molecular Dynamics Study of Binding of Μ-Conotoxin GIIIA to the Voltage-Gated Sodium Channel Na(v)1.4. PLoS ONE.

[126] Cummins T.R., Aglieco F., Dib-Hajj S.D. (2002). Critical Molecular Determinants of Voltage-Gated Sodium Channel Sensitivity to Mu-Conotoxins GIIIA/B. Mol. Pharmacol..

[127] Chow C.Y., Chin Y.K.-Y., Walker A.A., Guo S., Blomster L.V., Ward M.J., Herzig V., Rokyta D.R., King G.F. (2020). Venom Peptides with Dual Modulatory Activity on the Voltage-Gated Sodium Channel NaV1.1 Provide Novel Leads for Development of Antiepileptic Drugs. ACS Pharmacol. Transl. Sci..

[128] Wu Q., Huang J., Fan X., Wang K., Jin X., Huang G., Li J., Pan X., Yan N. (2023). Structural Mapping of Nav1.7 Antagonists. Nat. Commun..

[129] Catterall W.A. (1986). Molecular Properties of Voltage-Sensitive Sodium Channels. Annu. Rev. Biochem..

[130] Wu Y., Ma H., Zhang F., Zhang C., Zou X., Cao Z. (2018). Selective Voltage-Gated Sodium Channel Peptide Toxins from Animal Venom: Pharmacological Probes and Analgesic Drug Development. ACS Chem. Neurosci..

[131] Botte M., Huber S., Bucher D., Klint J.K., Rodríguez D., Tagmose L., Chami M., Cheng R., Hennig M., Abdul Rahman W. (2022). Apo and Ligand-Bound High Resolution Cryo-EM Structures of the Human Kv3.1 Channel Reveal a Novel Binding Site for Positive Modulators. PNAS Nexus.

[132] Torres Y.P., Morera F.J., Carvacho I., Latorre R. (2007). A Marriage of Convenience: Beta-Subunits and Voltage-Dependent K^+^ Channels. J. Biol. Chem..

[133] Jiang Y., Lee A., Chen J., Ruta V., Cadene M., Chait B.T., MacKinnon R. (2003). X-Ray Structure of a Voltage-Dependent K^+^ Channel. Nature.

[134] Long S.B., Campbell E.B., Mackinnon R. (2005). Crystal Structure of a Mammalian Voltage-Dependent Shaker Family K^+^ Channel. Science.

[135] Van Theemsche K.M., Van de Sande D.V., Snyders D.J., Labro A.J. (2020). Hydrophobic Drug/Toxin Binding Sites in Voltage-Dependent K^+^ and Na+ Channels. Front. Pharmacol..

[136] Sumino A., Sumikama T., Uchihashi T., Oiki S. (2019). High-Speed AFM Reveals Accelerated Binding of Agitoxin-2 to a K^+^ Channel by Induced Fit. Sci. Adv..

[137] Wu Y., Yan Y., Yang Y., Bian S., Rivetta A., Allen K., Sigworth F.J. (2025). CryoEM Structures of Kv1.2 Potassium Channels, Conducting and Non-Conducting. eLife.

[138] Gao Y., Xu S., Cui X., Xu H., Qiu Y., Wei Y., Dong Y., Zhu B., Peng C., Liu S. (2023). Molecular Insights into the Gating Mechanisms of Voltage-Gated Calcium Channel CaV2.3. Nat. Commun..

[139] Catterall W.A. (2011). Voltage-Gated Calcium Channels. Cold Spring Harb. Perspect. Biol..

[140] Catterall W.A. (2023). Voltage Gated Sodium and Calcium Channels: Discovery, Structure, Function, and Pharmacology. Channels.

[141] Ichida S., Abe J., Yu-an Z., Minami T., Wada T., Yazawa M., Sohma H. (2003). Structural Specificity for the Inhibitory Effect of Calmodulin on Specific 125I-Omega-Conotoxin GVIA Binding. Neurochem. Res..

[142] Yao X., Gao S., Yan N. (2023). Structural Biology of Voltage-Gated Calcium Channels. Channels.

[143] Lewis R.J., Garcia M.L. (2003). Therapeutic Potential of Venom Peptides. Nat. Rev. Drug Discov..

[144] Gao S., Yao X., Yan N. (2021). Structure of Human Cav2.2 Channel Blocked by the Painkiller Ziconotide. Nature.

[145] Nimmrich V., Gross G. (2012). P/Q-Type Calcium Channel Modulators: P/Q-Type Calcium Channel Blockers. Br. J. Pharmacol..

[146] Li Z., Cong Y., Wu T., Wang T., Lou X., Yang X., Yan N. (2024). Structural Basis for Different ω-Agatoxin IVA Sensitivities of the P-Type and Q-Type Cav2.1 Channels. Cell Res..

[147] Gao S., Yao X., Chen J., Huang G., Fan X., Xue L., Li Z., Wu T., Zheng Y., Huang J. (2023). Structural Basis for Human Cav1.2 Inhibition by Multiple Drugs and the Neurotoxin Calciseptine. Cell.

[148] McGivern J.G. (2007). Ziconotide: A Review of Its Pharmacology and Use in the Treatment of Pain. Neuropsychiatr. Dis. Treat..

[149] Ohashi N., Uta D., Ohashi M., Hoshino R., Baba H. (2024). Omega-Conotoxin MVIIA Reduces Neuropathic Pain after Spinal Cord Injury by Inhibiting N-Type Voltage-Dependent Calcium Channels on Spinal Dorsal Horn. Front. Neurosci..

[150] Tarcha E.J., Olsen C.M., Probst P., Peckham D., Muñoz-Elías E.J., Kruger J.G., Iadonato S.P. (2017). Safety and Pharmacodynamics of Dalazatide, a Kv1.3 Channel Inhibitor, in the Treatment of Plaque Psoriasis: A Randomized Phase 1b Trial. PLoS ONE.

[151] Wang X., Li G., Guo J., Zhang Z., Zhang S., Zhu Y., Cheng J., Yu L., Ji Y., Tao J. (2019). Kv1.3 Channel as a Key Therapeutic Target for Neuroinflammatory Diseases: State of the Art and Beyond. Front. Neurosci..

[152] Bergmann R., Kubeil M., Zarschler K., Chhabra S., Tajhya R.B., Beeton C., Pennington M.W., Bachmann M., Norton R.S., Stephan H. (2017). Distribution and Kinetics of the Kv1.3-Blocking Peptide HsTX1[R14A] in Experimental Rats. Sci. Rep..

[153] Rashid M.H., Huq R., Tanner M.R., Chhabra S., Khoo K.K., Estrada R., Dhawan V., Chauhan S., Pennington M.W., Beeton C. (2014). A Potent and Kv1.3-Selective Analogue of the Scorpion Toxin HsTX1 as a Potential Therapeutic for Autoimmune Diseases. Sci. Rep..

[154] Peigneur S., Orts D., Prieto da Silva A., Oguiura N., Boni-Mitake M., Brandt de Oliveira E., Zaharenko A.J., de Freitas J.C., Tytgat J. (2012). 13. Crotamine Pharmacology Revisited: Novel Insights Based on the Inhibition of Kv Channels. Toxicon.

[155] Lee S., Kim Y., Back S.K., Choi H.-W., Lee J.Y., Jung H.H., Ryu J.H., Suh H.-W., Na H.S., Kim H.J. (2010). Analgesic Effect of Highly Reversible ω-Conotoxin FVIA on N Type Ca^2+^ Channels. Mol. Pain.

[156] Beeton C., Wulff H., Barbaria J., Clot-Faybesse O., Pennington M., Bernard D., Cahalan M.D., Chandy K.G., Béraud E. (2001). Selective Blockade of T Lymphocyte K(+) Channels Ameliorates Experimental Autoimmune Encephalomyelitis, a Model for Multiple Sclerosis. Proc. Natl. Acad. Sci. USA.

[157] Weuring W.J., Singh S., Volkers L., Rook M.B., van ’t Slot R.H., Bosma M., Inserra M., Vetter I., Verhoeven-Duif N.M., Braun K.P.J. (2020). NaV1.1 and NaV1.6 Selective Compounds Reduce the Behavior Phenotype and Epileptiform Activity in a Novel Zebrafish Model for Dravet Syndrome. PLoS ONE.

[158] Richards K.L., Milligan C.J., Richardson R.J., Jancovski N., Grunnet M., Jacobson L.H., Undheim E.A.B., Mobli M., Chow C.Y., Herzig V. (2018). Selective NaV1.1 Activation Rescues Dravet Syndrome Mice from Seizures and Premature Death. Proc. Natl. Acad. Sci. USA.

[159] Ferrari S., Di Iorio E., Barbaro V., Ponzin D., Sorrentino F.S., Parmeggiani F. (2011). Retinitis Pigmentosa: Genes and Disease Mechanisms. Curr. Genomics.

[160] Pennington K.L., DeAngelis M.M. (2016). Epidemiology of Age-Related Macular Degeneration (AMD): Associations with Cardiovascular Disease Phenotypes and Lipid Factors. Eye Vis..

[161] Tochitsky I., Kienzler M.A., Isacoff E., Kramer R.H. (2018). Restoring Vision to the Blind with Chemical Photoswitches. Chem. Rev..

[162] Drivas T.G., Bennett J. (2012). The Bionic Retina: A Small Molecule with Big Potential for Visual Restoration. Neuron.

[163] Busskamp V., Picaud S., Sahel J.A., Roska B. (2012). Optogenetic Therapy for Retinitis Pigmentosa. Gene Ther..

[164] Yan B., Viswanathan S., Brodie S.E., Deng W.-T., Coleman K.E., Hauswirth W.W., Nirenberg S. (2023). A Clinically Viable Approach to Restoring Visual Function Using Optogenetic Gene Therapy. Mol. Ther. Methods Clin. Dev..

[165] Ong J.M., da Cruz L. (2012). A Review and Update on the Current Status of Stem Cell Therapy and the Retina. Br. Med. Bull..

[166] Nazari H., Zhang L., Zhu D., Chader G.J., Falabella P., Stefanini F., Rowland T., Clegg D.O., Kashani A.H., Hinton D.R. (2015). Stem Cell Based Therapies for Age-Related Macular Degeneration: The Promises and the Challenges. Prog. Retin. Eye Res..

[167] da Cruz L., Fynes K., Georgiadis O., Kerby J., Luo Y.H., Ahmado A., Vernon A., Daniels J.T., Nommiste B., Hasan S.M. (2018). Phase 1 Clinical Study of an Embryonic Stem Cell-Derived Retinal Pigment Epithelium Patch in Age-Related Macular Degeneration. Nat. Biotechnol..

[168] Ramirez K.A., Drew-Bear L.E., Vega-Garces M., Betancourt-Belandria H., Arevalo J.F. (2023). An Update on Visual Prosthesis. Int. J. Retina Vitreous.

[169] Fujikado T., Kamei M., Sakaguchi H., Kanda H., Endo T., Hirota M., Morimoto T., Nishida K., Kishima H., Terasawa Y. (2016). One-Year Outcome of 49-Channel Suprachoroidal-Transretinal Stimulation Prosthesis in Patients with Advanced Retinitis Pigmentosa. Investig. Ophthalmol. Vis. Sci..

[170] Terasawa Y., Tashiro H., Nakano Y., Ohta J. (2018). Safety and Efficacy of Semichronic Suprachoroidal Transretinal Stimulation with Femtosecond Laser-Induced Porosity and Smooth-Surface Electrodes. Sens. Mater..

[171] Tashiro H., Kuwabara M., Nakano Y., Terasawa Y., Osawa K., Yoshimura Y., Doi H., Ohta J. (2018). In Vitro and in Vivo Long-Term Electrochemical Properties of Electrodes with Femtosecond-Laser-Induced Porosity for Visual Prostheses Based on Suprachoroidal Transretinal Stimulation. Sens. Mater..

[172] Nomura S., Tashiro H., Terasawa Y., Nakano Y., Haruta M., Sasagawa K., Takehara H., Morimoto T., Fujikado T., Ohta J. (2023). Effects of Long-Term in Vivo Stimulation on the Electrochemical Properties of a Porous Stimulation Electrode for a Suprachoroidal–Transretinal Stimulation (STS) Retinal Prosthesis. Sens. Mater..

[173] Tochitsky I., Kramer R.H. (2015). Optopharmacological Tools for Restoring Visual Function in Degenerative Retinal Diseases. Curr. Opin. Neurobiol..

[174] Tochitsky I., Helft Z., Meseguer V., Fletcher R.B., Vessey K.A., Telias M., Denlinger B., Malis J., Fletcher E.L., Kramer R.H. (2016). How Azobenzene Photoswitches Restore Visual Responses to the Blind Retina. Neuron.

[175] Fortin D.L., Banghart M.R., Dunn T.W., Borges K., Wagenaar D.A., Gaudry Q., Karakossian M.H., Otis T.S., Kristan W.B., Trauner D. (2008). Photochemical Control of Endogenous Ion Channels and Cellular Excitability. Nat. Methods.

[176] Mourot A., Tochitsky I., Kramer R.H. (2013). Light at the End of the Channel: Optical Manipulation of Intrinsic Neuronal Excitability with Chemical Photoswitches. Front. Mol. Neurosci..

[177] Ko K.W., Rasband M.N., Meseguer V., Kramer R.H., Golding N.L. (2016). Serotonin Modulates Spike Probability in the Axon Initial Segment through HCN Channels. Nat. Neurosci..

[178] Mourot A., Kienzler M.A., Banghart M.R., Fehrentz T., Huber F.M.E., Stein M., Kramer R.H., Trauner D. (2011). Tuning Photochromic Ion Channel Blockers. ACS Chem. Neurosci..

[179] Tochitsky I., Polosukhina A., Degtyar V.E., Gallerani N., Smith C.M., Friedman A., Van Gelder R.N., Trauner D., Kaufer D., Kramer R.H. (2014). Restoring Visual Function to Blind Mice with a Photoswitch That Exploits Electrophysiological Remodeling of Retinal Ganglion Cells. Neuron.

[180] Tochitsky I., Trautman J., Gallerani N., Malis J.G., Kramer R.H. (2017). Restoring Visual Function to the Blind Retina with a Potent, Safe and Long-Lasting Photoswitch. Sci. Rep..

[181] Casson R.J., Daniels E., Barras C., Dwyer A., Strem B., Wykoff C.C., Van Gelder R.N. (2024). Synthetic Phototransduction with a Light-Responsive Molecule (KIO-301) in Advanced Retinitis Pigmentosa: The ABACUS-1 Phase 1/2 Trial. Investig. Ophthalmol. Vis. Sci..

[182] Terlau H., Olivera B.M. (2004). Conus Venoms: A Rich Source of Novel Ion Channel-Targeted Peptides. Physiol. Rev..

[183] Catterall W.A., Lenaeus M.J., Gamal El-Din T.M. (2020). Structure and Pharmacology of Voltage-Gated Sodium and Calcium Channels. Annu. Rev. Pharmacol. Toxicol..

[184] Remme C.A. (2023). SCN5A Channelopathy: Arrhythmia, Cardiomyopathy, Epilepsy and Beyond. Philos. Trans. R. Soc. Lond. B Biol. Sci..

[185] Wisedchaisri G., Gamal El-Din T.M. (2022). Druggability of Voltage-Gated Sodium Channels-Exploring Old and New Drug Receptor Sites. Front. Pharmacol..

[186] Sahel J.-A., Boulanger-Scemama E., Pagot C., Arleo A., Galluppi F., Martel J.N., Esposti S.D., Delaux A., de Saint Aubert J.-B., de Montleau C. (2021). Partial Recovery of Visual Function in a Blind Patient after Optogenetic Therapy. Nat. Med..

[187] Cordeiro S., Finol-Urdaneta R.K., Köpfer D., Markushina A., Song J., French R.J., Kopec W., de Groot B.L., Giacobassi M.J., Leavitt L.S. (2019). Conotoxin ΚM-RIIIJ, a Tool Targeting Asymmetric Heteromeric Kv1 Channels. Proc. Natl. Acad. Sci. USA.

[188] Yotsu-Yamashita M., Kim Y.H., Dudley S.C., Choudhary G., Pfahnl A., Oshima Y., Daly J.W. (2004). The Structure of Zetekitoxin AB, a Saxitoxin Analog from the Panamanian Golden Frog Atelopus Zeteki: A Potent Sodium-Channel Blocker. Proc. Natl. Acad. Sci. USA.

[189] Park E.R., Denomme N., Hajare H.S., Du Bois J. (2025). A Chemogenetic Ligand-Receptor Pair for Voltage-Gated Sodium Channel Subtype-Selective Inhibition. bioRxiv.

